# Natural *Green* Coating Inhibits Adhesion of Clinically Important Bacteria

**DOI:** 10.1038/srep08287

**Published:** 2015-02-06

**Authors:** Danielle S. Trentin, Denise B. Silva, Amanda P. Frasson, Olena Rzhepishevska, Márcia V. da Silva, Elinor de L. Pulcini, Garth James, Gabriel V. Soares, Tiana Tasca, Madeleine Ramstedt, Raquel B. Giordani, Norberto P. Lopes, Alexandre J. Macedo

**Affiliations:** 1 Faculdade de Farmácia, Universidade Federal do Rio Grande do Sul, Porto Alegre/RS, 90610-000, Brazil; 2 Centro de Biotecnologia, Universidade Federal do Rio Grande do Sul, Porto Alegre/RS, 91501-970, Brazil; 3 Núcleo de Pesquisas em Produtos Naturais e Sintéticos (NPPNS), Faculdade de Ciências Farmacêuticas de Ribeirão Preto, Universidade de São Paulo, Ribeirão Preto/SP, 14040-903, Brazil; 4 Department of Chemistry, Umeå University, Umeå, SE-90187, Sweden; 5 Centro de Ciências Biológicas and Departamento de Bioquímica, Universidade Federal de Pernambuco, Recife/PE, 50670-901, Brazil; 6 Center for Biofilm Engineering, Montana State University, Bozeman/MT, 59717, United States of America; 7 Instituto de Física, Universidade Federal do Rio Grande do Sul, Porto Alegre/RS, 91509-900, Brazil; 8 Centro de Ciências da Saúde, Departamento de Farmácia, Universidade Federal do Rio Grande do Norte, Natal/RN, 59010-180, Brazil

## Abstract

Despite many advances, biomaterial-associated infections continue to be a major clinical problem. In order to minimize bacterial adhesion, material surface modifications are currently being investigated and natural products possess large potential for the design of innovative surface coatings. We report the bioguided phytochemical investigation of *Pityrocarpa moniliformis* and the characterization of tannins by mass spectrometry. It was demonstrated that B-type linked proanthocyanidins-coated surfaces, here termed *Green* coatings, reduced Gram-positive bacterial adhesion and supported mammalian cell spreading. The proposed mechanism of bacterial attachment inhibition is based on electrostatic repulsion, high hydrophilicity and the steric hindrance provided by the coating that blocks bacterium-substratum interactions. This work shows the applicability of a prototype *Green*-coated surface that aims to promote necessary mammalian tissue compatibility, while reducing bacterial colonization.

Biomaterials play a central role in management of diseases and in improvement of healthcare. As the population ages, there is a growing need to sustain functions and physiological processes critical to life by the use of biomaterials^1^,^2^,^3^. Surfaces of an implanted material must present compatibility with the surrounding tissue and, at the same time, it must prevent pathogenic microbial adhesion, thereby acting as a dual-function surface^4^. Once a suitable environment for attachment is established, bacterial species can adhere to the surface and proliferate as biofilms – microbial aggregates encased in a protective self-produced extracellular matrix^5^. Importantly, biofilm formation promotes metabolic, phenotypic and genotypic changes, which increase the resistance of bacteria to antimicrobial agents up to 1000-fold compared to planktonic bacteria, making eradication of bacteria in biofilms extremely difficult^6^. For these reasons, adhesion – the first stage of biofilm formation – can be considered an attractive target to restrict or to control biofilm infections.

Intensive efforts have been focused on the elimination or substantial reduction of bacterial attachment and biofilm formation on biomaterials. Currently, modifications of surfaces via surface-engineering methods to produce antifouling materials^7^,^8^,^9^,^10^ as well strategies involving materials with immobilized antibiotics or antibiotic eluting materials with site-localized delivery^11^,^12^,^13^ are being explored. Although promising results have been demonstrated with antimicrobial agents, their continuous use on surfaces may select and enhance the emergence of new resistant bacterial strains^14^,^15^. In this sense, advanced approaches that do not necessarily rely on classic antibiotic mechanisms are being developed to create new materials, including (i) materials that release biofilm dispersal agents; (ii) bacteriophage-releasing materials; (iii) the use of probiotic bacteria to protect materials from pathogenic infection; (iv) the application of antisense peptide nucleic acids to interfere with bacterial gene expression involved in biofilm formation, as a gene therapy of implant infections; and (v) through antibiofilm surface modifications and coatings^16^,^17^. A new generation of materials mimics the nanotopography of natural surfaces, such as insect wings, shark skin, and lotus leaves, which naturally exhibit antifouling properties and prevent particles, algal spores, and bacterial cells from attaching to their surfaces^18^,^19^. However, the use of natural products to control biointerfacial interactions and to design anti-infective properties that are compatible with the surrounding cell environment is still scarce.

In this study we purified and elucidated condensed tannins, not previously described, from the Brazilian medicinal plant *Pityrocarpa moniliformis*, and demonstrated that they act as anti-adhesive substances, both in solution and as immobilized on a surface, against a range of Gram-positive bacteria (*Staphylococccus epidermidis, Staphylococcus aureus* and *Enterococcus faecalis*). Since these B-type proanthocyanidins prevent bacterial adhesion without killing cells – thereby reducing the adaptive development of resistant strains - we develop a prototype of natural product-coated surfaces, hereafter called *Green* coating, which repelled the Gram-positive strains tested while allowing compatibility with mammalian cells.

## Results

### Bioguided fractionation revealed the B-type linked proanthocyanidins as the bioactive compounds

**Figure 1:**
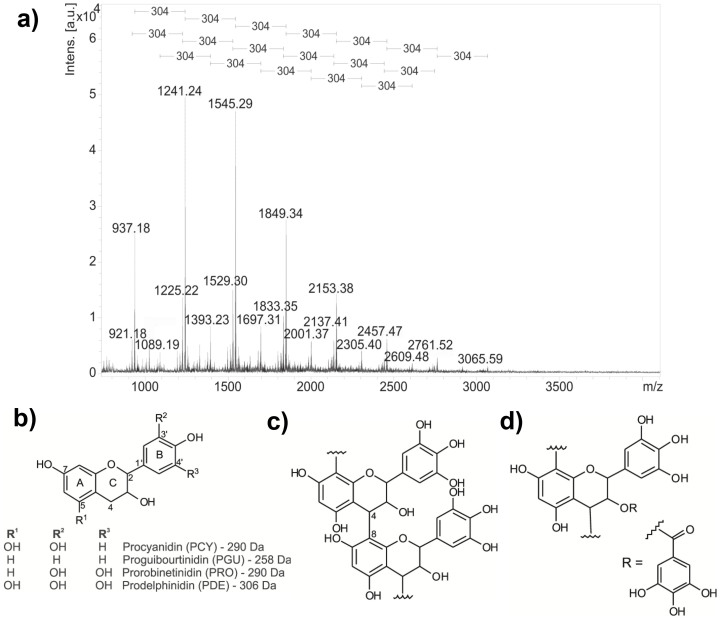
Proanthocyanidins from *P. moniliformis* leaves. (a) Mass spectra (positive ionization mode) of PMP, highlighting the consecutive mass differences of 304 Da in the polymeric series; (b) chemical structures of proanthocyanidin repeating units: procyanidin (PCY), proguibourtinidin (PGU), prorobinetinidin (PRO) and prodelphinidin (PDE); (c) typical linear proanthocyanidins possessing B-type linkage and (d) the proanthocyanidin with galloyl unit (*m/z* 785 [M + Na]^+^) identified in series C from PMP.

After screening for antibiofilm activity against *S. epidermidis* (Supplementary Fig. S1) and elucidating all *P. moniliformis* fractions generated through bioguided fractionation (Supplementary Data and Discussion 1 and 2, Supplementary Table S1–S3 and Supplementary Fig. S2–S9), we focused on the most yielded and active fraction 7, named PMP (*P. moniliformis* proanthocyanidins). Proanthocyanidins, or condensed tannins, are biomacromolecules produced through polymerization of flavan-3-ol repeating units, generally composed of complex mixtures of oligomeric structures. Concerning the interflavanyl bond nature, B-type proanthocyanidins show the linkage between monomers through the C-4 of the top unit and the C-6 or C-8 of the terminal unit, while the A-type proanthocyanidins additionally possess one ether type bond between the C-2 position of the top unit and the hydroxyl unit at the C-5 or C-7 of the terminal residue^20^,^21^. PMP was characterized by MALDI-MS and MS/MS analysis as series of polyflavan-3-ol oligomers that have repeating units with a mass difference of 304 Da (Fig. 1a and Supplementary Table S1), indicating coupling of prodelphinidin units through B-type linkages (Fig. 1b and c; data and MS/MS spectrum for each oligomer are in Supplementary Table S2 and Supplementary Fig. S3–S7). PMP contains three distinct polymeric series (initial ions: series A – *m/z* 937, series B – *m/z* 921 and series C – *m/z* 1089), which are composed of prodelphinidin units (up to 10) and only one unit of procyanidin, prorobinetinidin or galloyl group (Fig. 1d and Supplementary Table S1) linked to prodelphinidin units, respectively to series A, B and C.

### Proanthocyanidins had non-biocidal antibiofilm action and were non-toxic to mammalian cell at lower concentrations

**Figure 2:**
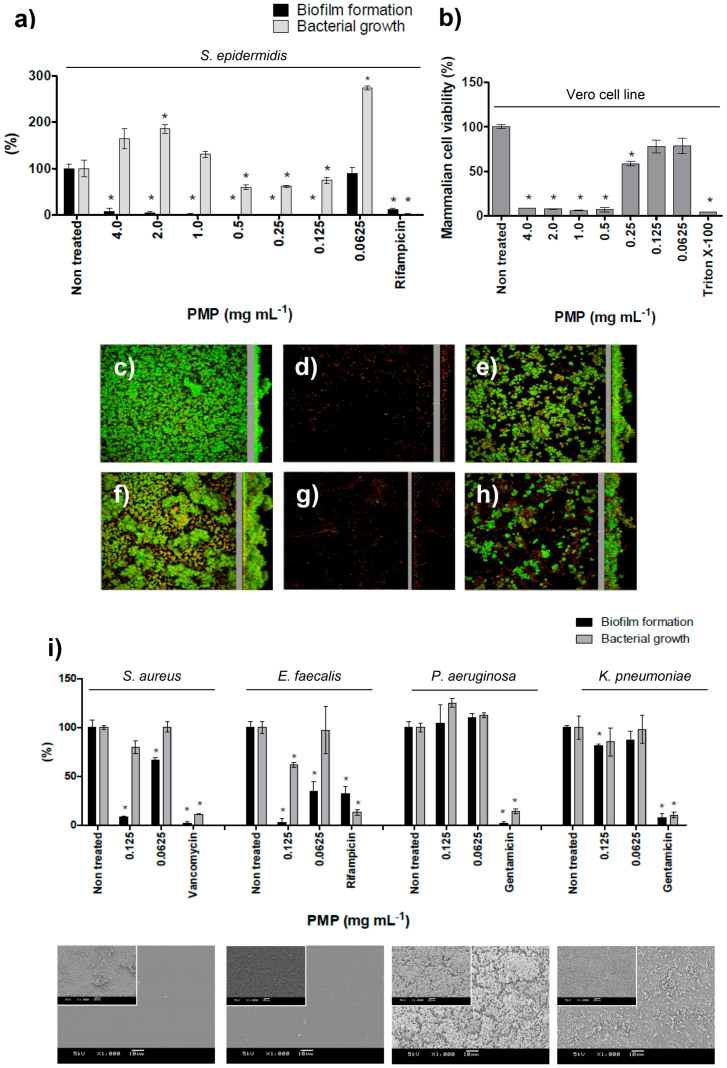
The effect of different concentrations of PMP on bacterial biofilm formation and growth, and on viability of epithelial mammalian cells. (a) Dose-response curve of PMP tested against *S. epidermidis* biofilm formation and bacterial growth. (b) Dose-response curve of PMP tested upon mammalian cells viability. The biofilm topography of non-treated *S. epidermidis* on (c) polystyrene and on (f) glass surfaces; the exposition of *S. epidermidis* to 0.125 mg mL^−1^ of PMP (d and g, respectively to polystyrene and glass) and the biofilm formation when cells were exposed to 0.0625 mg mL^−1^ (e and h, respectively to polystyrene and glass). CLSM methodology can be found in Supplementary Methods. (i) Effect of PMP on other bacterial species involved in implant infections. SEM images show bacteria after exposure to 0.125 mg mL^−1^ and inserts present the respective non-treated control (bars indicate 10 μm). * represents statistical difference (p < 0.05) between treated and non-treated samples when analyzed by Student's *t*-test.

When in solution, PMP completely suppressed biofilm formation by *S. epidermidis* from 4.0 mg mL^−1^ down to 0.125 mg mL^−1^ while bacterial growth was influenced to a much lesser extent (Fig. 2a). Confocal microscopy images obtained using LIVE/DEAD staining (Fig. 2c–h) showed the disappearance of the architectural features of untreated *S. epidermidis* biofilm after exposure to PMP on both hydrophobic (polystyrene) and hydrophilic (glass) surfaces. The images correlated with the dose-response curve showing that at 0.125 mg mL^−1^ the PMP inhibited biofilm formation and kept most cells in the planktonic state, consequently most of them were removed during the washing procedure for the microscopy samples (Fig. 2d,g). At 0.0625 mg mL^−1^, *S. epidermidis* were able to form biofilms restoring their typical topography on both model surfaces (Fig. 2e,h). In addition, PMP did not demonstrate toxicity against an epithelial mammalian cell at the lowest concentration which prevented biofilm formation (0.125 mg mL^−1^, Fig. 2b).

In order to investigate the effect of PMP upon other bacterial species relevant for infections associated with indwelling medical devices, we analyzed four additional bacteria in the study (Fig. 2i) and verified that PMP was active against the Gram-positives *S. aureus* and *E. faecalis*, at 0.125 mg mL^−1^. However, PMP was not able to prevent adhesion of the Gram-negatives *P. aeruginosa* and *K. pneumoniae*.

### In solution, proanthocyanidins altered bacterial surface hydrophobicity and prevented the attachment of bacteria and non-biological particles

**Figure 3:**
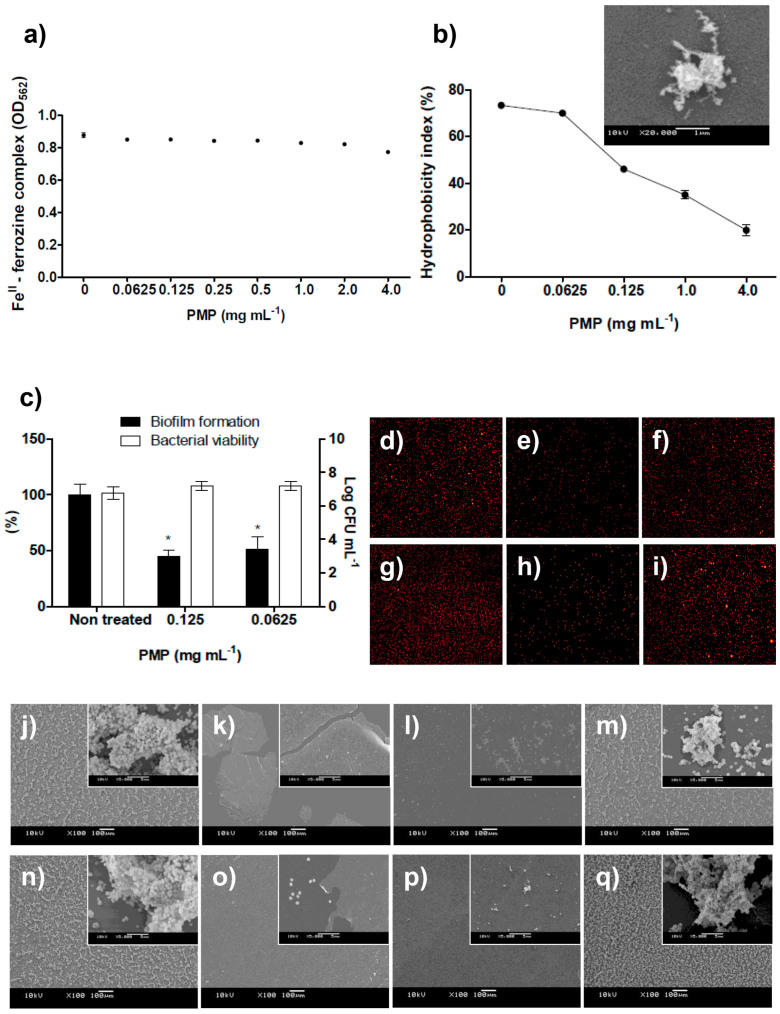
(a) The concentration of ferrozine-Fe^II^ complex was not decreased in the presence of PMP, indicating that these proanthocyanidins are not strong Fe^II^ chelators (the standard curve established to determine the Fe^II^ concentration to be used in the ferrozine assay and the curve of positive-chelator 2,2-bipyridyl can be found in Supplementary Fig. S10). (b) The dose-dependent decreasing of *S. epidermidis* surface hydrophobicity index (HBPI); values of HPBI greater than 70% indicated hydrophobic bacterial surface (non-treated or treated with PMP at 0.0625 mg mL^−1^); less than 70% indicated hydrophilic bacterial surface. SEM image shows that surface of *S. epidermidis* cells become covered by amorphous material in solutions containing PMP. (c) *S. epidermidis* partially recovered the ability to form biofilm and remained viable after exposure to proanthocyanidins for 24 h with three subsequent washes using sterile 0.9% NaCl. * represents statistical difference (p < 0.05) between treated and non-treated samples when analyzed by Student's *t*-test. (d–i) Fluorescence microscopy images (10x magnification) demonstrated that (d and g) non-treated microspheres and (f and i) microspheres treated with 0.0625 mg mL^−1^ of PMP attach to Permanox and to glass surfaces, respectively, while attachment was inhibited for spheres exposed to 0.125 mg mL^−1^ of PMP (e and h, respectively for Permanox and glass surfaces), similarly as observed for *S. epidermidis* cells treated with PMP. similarly as observed for *S. epidermidis* cells treated with PMP. (j–q) SEM images of *S. epidermidis* treated with PMP. Untreated *S. epidermidis* displayed several microcolonies on (j) Permanox and (n) on glass surfaces. When *S. epidermidis* was exposed to 4.0 mg mL^−1^ of PMP, there was no sign of attached cells while some regions of (k) Permanox and (o) glass substrates, and PMP spontaneously adhered on hydrophobic and hydrophilic substrates (note the insert showing that single cells adhered where there is no PMP film). Inhibition of biofilm formation at 0.125 mg mL^−1^ of PMP is observed on (l) Permanox and on (p) glass surfaces. The ability of *S. epidermidis* to form protective biofilm is not inhibited by 0.0625 mg mL^−1^ of PMP neither on (m) Permanox nor on (q) glass substrates. Bars represent 100 μm and in the inserts, 5 μm. All of these methodologies can be found in Supplementary Methods.

The investigation of how PMP prevents bacterial adhesion provided insights into the mechanism of action of proanthocyanidins and led to several findings: (i) Iron depletion (chelation) is not likely involved in PMP bioactivity (Fig. 3a, Supplementary Data and Discussion 3 and Supplementary Fig. S10). (ii) The bacterial surface hydrophobicity, governing bacterial attachment to abiotic surfaces^22^,^23^,^24^ is progressively altered following the concentration of PMP, which could partially explain the anti-adhesive effect observed especially since the hydrophilic character increased substantially among active and non-active concentrations (Fig. 3b). In scanning electron microscopy (SEM) images, amorphous material is seen around (on top of) bacterial cells exposed to PMP demonstrating formation of some type of hydrophilic covering layer on the bacterial surface (Fig. 3b). It is known that complexation of proteins by polyphenols (such as PMP) led to the formation of insoluble aggregates that can be visualized by SEM images^25^. Therefore, we hypothesized that in our study a coaggregation of proanthocyanidins, proteins from the culture medium and bacterial cells takes place. (iii) After treating bacteria with PMP for 24 h and then washing to remove the agent, *S. epidermidis* cells remained viable but the ability to form biofilm was not completely restored (Fig. 3c), indicating substantial changes in the adhesive properties of the cells and suggesting that the covering layer formed on the bacterial cell surface withstood washing. (iv) To better understand the anti-adhesive effect of PMP, bacterial cells were replaced by fluorescent microspheres having similar features to *S. epidermidis*, including size (1 μm) and a hydrophobic and anionic surface. Since abiotic microspheres were also prevented from adhering to the same extent as bacterial cells (Fig. 3d–i), physiological pathways alone could not explain the anti-adhesion effect. (v) Finally, PMP is able to self-adhere on hydrophobic and hydrophilic abiotic surfaces, producing a film that prevents bacterial attachment (Fig. 3j–q). This ability to self-adhere to surfaces led us to explore proanthocyanidins as potential surface-coating agents.

### *Green*-coated surfaces presented hydrophilic character and had a film thickness in the nanometer range

**Figure 4:**
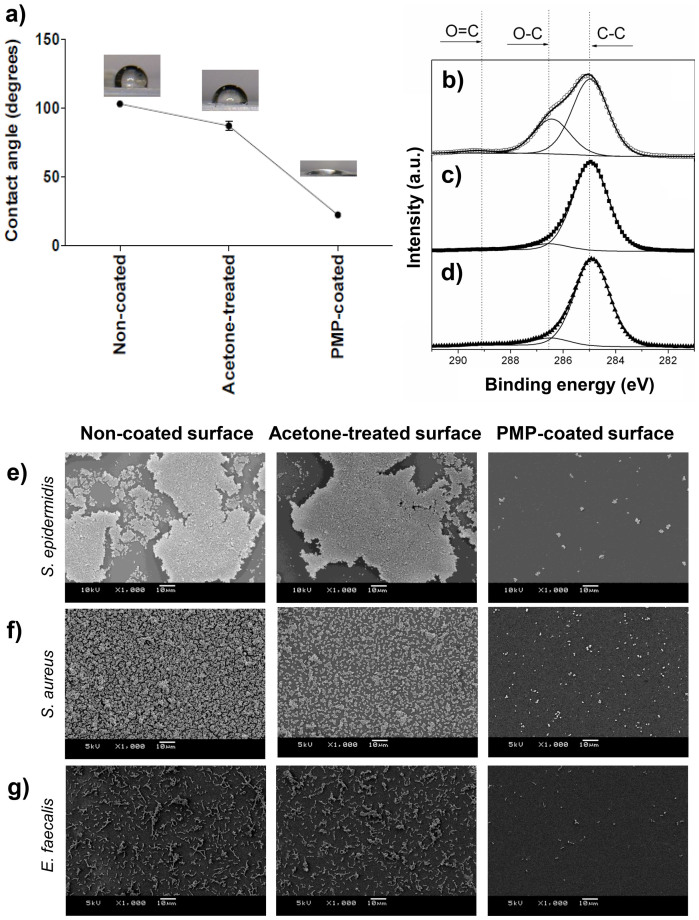
*Green*-coated surface analyses. (a) WCA of non-coated, acetone-treated and PMP coated-surfaces. (b) XPS analyses of PMP coated-surfaces, (c) acetone-treated and (d) non-coated surfaces. SEM images of adhesion and biofilm formation by (e) *S. epidermidis*, (f) *S. aureus* and (g) *E. faecalis* on non-coated surface, acetone-treated surface and PMP-coated surface, respectively. Bars indicate 10 μm.

The prototype *Green*-coated surfaces, produced by spin-coating, had a highly hydrophilic character, with a water contact angle (WCA) of about 20°, while non-coated and 70% acetone (PMP diluent)-treated Permanox surfaces were hydrophobic, WCA of 100° and 85°, respectively (Fig. 4a). XPS analyses of PMP coated-samples showed an increase in the relative concentration of the C-O (286.5 eV) component (Fig. 4b) when compared to control samples (Fig. 4c,d). This result indicates the presence of prodelphinidin oligomers in the coating, since they contain a high number of polar C-O bonds, which also explains the increase in hydrophilicity. Using ellipsometry, the thickness of the PMP film on Permanox slides was determined to be 39 ± 13 nm. Acetone treatment alone of Permanox slides did not result in any significant change in thickness. Likewise, XPS spectra for non-coated (Fig. 4d) and acetone-treated (Fig. 4c) samples were very similar showing one component at 284.9 eV attributed to C-C bonds in the Permanox slide and some C-O bonds attributed to surface oxidation or possible contamination during the manufacturing process.

### *Green* coating supports mammalian cell spreading while exhibiting anti-adherent properties for Gram-positive bacteria

The *Green*-coated surfaces were resistant to bacterial adhesion, presenting only few cell clusters or just single attached cells (Fig. 4e–g). Non-coated and acetone-treated surfaces enabled bacterial adherence and accumulation, allowing biofilm formation (Fig. 4e–g). Non-adherent cells (planktonic cells) were viable after exposure to the surfaces, showing that the anti-attachment effect is not due to a reduction of viable bacteria in solution (Supplementary Fig. S11).

The strength of attachment to the substratum of the PMP coating was investigated by exposing the specimens to three different conditions, followed by evaluation of remaining antibiofilm activity (Supplementary Fig. S11 and Supplementary Methods). SEM images show that antibiofilm activity is gradually lost according the time exposed to a 0.9% NaCl physiologic solution. After splash washing, the coating kept most of its antibiofilm activity compared with to non-washed coatings. However, after 24 h of soaking in 0.9% NaCl physiologic solution, the number of bacteria able to attach increased and following 48 h of soaking, the antibiofilm effect was lost, allowing *S. epidermidis* to colonize the surface and produce biofilm. Again, the viability of planktonic cells was not negatively affected by the surface at any of these time points (Supplementary Fig. S11).

**Figure 5:**
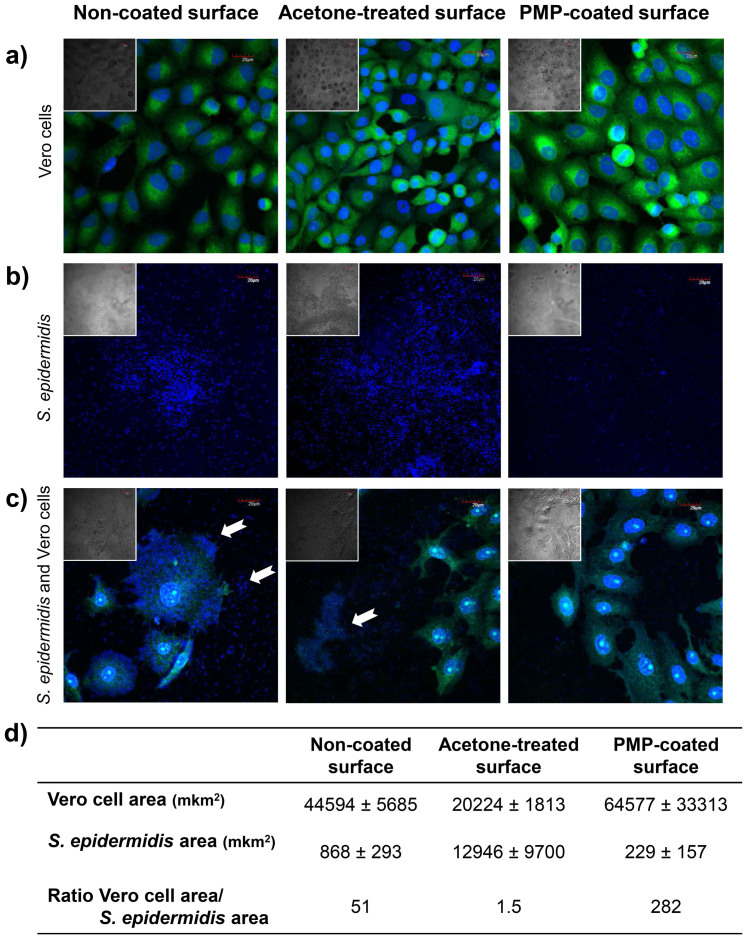
Co-culture of bacteria and mammalian cells using the perioperative bacterial model^26^. (a) Adhesion and spreading of mammalian Vero cells, (b) adhesion of *S. epidermidis*, (c) co-culture of *S. epidermidis* and mammalian Vero cells on non-coated, acetone-treated and PMP-coated surfaces, respectively; and (d) quantitative data of co-culture experiments as measurements of mammalian and bacterial cell area. Samples were imaged by 60x CLSM (FLUTAX-2 labels microtubules and DAPI stains DNA) and by differential interference contrast (DIC – in the inserts). White arrows indicate bacterial cell clusters. Bars in the images indicate 20 μm.

Although the mammalian cells only remained fully viable at the lower active concentration of PMP in solution (Fig. 2b), their adhesion on the *Green*-coated surfaces was very similar to the controls (Fig. 5a), indicating biocompatibility between mammalian cells and immobilized proanthocyanidins.

During infection associated with medical devices, interaction of bacteria and mammalian cells with the medical device takes place. In order to evaluate the competition for available *Green*-coating surface between mammalian cells and bacteria, we designed a co-culture experiment where bacteria are allowed to adhere prior to cell seeding, according the perioperative bacterial contamination model. CLSM images demonstrate that Vero cells continued to attach in the presence of a reduced number of adhering staphylococci, when compared to the control surfaces - which present higher number of bacteria, including cell clusters (Fig. 5b and c). The quantitative analyses of these data confirmed that *Green*-coated surfaces displayed the best performance during the co-culture challenge, since presented the highest ratio between the mammalian cells and the bacteria cells, when compared with controls (Fig. 5d).

## Discussion

In contrast to classical antibiotics that generally kill bacteria, our results show that PMP modifies the process of bacterial adhesion by changing the characteristics of the surfaces involved, preventing bacterial attachment both when the compound is in solution and when it is coating material surface. By reducing bacterial adhesion, PMP minimizes biofilm formation and forces bacteria to remain planktonic, a bacterial lifestyle that should be easier for the innate immune system and traditional antibiotics to clear than biofilms^27^.

Our findings show that the higher ratio between proanthocyanidins and flavonoids in *P. moniliformis* fractions results in better antibiofilm activity (Supplementary Data and Discussion 2) and B-type linked proanthocyanidins are the compounds responsible for the inhibition of biofilm formation by the Gram-positive bacteria. Previously, antiadherence and antibiofilm properties against Gram-negative *Escherichia coli* were assigned to proanthocyanidins derived from cranberry (*Vaccinium macrocarpon*)^28^,^29^,^30^,^31^,^32^,^33^, and their preventive effect in urinary tract infections has been clinically demonstrated^34^,^35^. Cranberry proanthocyanidins consist predominantly of procyanidin units^28^ and the oligomers containing at least one A-type interflavan linkage account for more than 91% of the oligomers^29^. The significance of the linkage type was emphasized by Howell and co-workers^30^ who found that proanthocyanidins containing A-type linkages displayed *E. coli* anti-adhesive activity compared to proanthocyanidins with only B-type linkages. The data presented by Howell *et al*^30^ and the difference in efficacy shown in Fig. 2i, where the biofilm formation by Gram-negative bacteria were not affected by PMP, led us to propose that there could be a difference in effectiveness of proanthocyanidins possessing A-type linkage, active against Gram-negative, compared to the B-type linkage, active against Gram-positive bacteria. Additionally, we might attribute the differential response exhibited for PMP against the Gram-positive and –negative strains to differences in the cell wall composition of these microorganisms, where linear B-type linked proanthocyanidins might better interact with the rigid peptidoglycan (Gram-positive) than the outer membrane (Gram-negative).

The dual-function presented by the prototype *Green*-coated surfaces showed (i) a strong reduction in the Gram-positive bacterial adhesion and biofilm formation and (ii) allowed adhesion and spreading of mammalian cells, combining two much desired features for a biomaterial. When challenged in the co-culture experiments, following the concept of “the race for the surface” between bacteria and mammalian cells^36^, the *Green*-coated surface better supported spreading of Vero cells while preventing *S. epidermidis* adhesion to a larger extent, compared to the control surfaces (Fig. 5). If a coating favors colonization of host cells over bacterial cells, the risk of infection decreases following host tissue integration^4^. It is important to take in account that peri-biomaterial tissue can acts as source of microorganism capable to provoke biomaterial-associated infection^37^. In this context, anti-adhesive materials are of great value since will reduce microbial biofilm formation on the “foreign” device and prevent it from becoming an additional stronghold for bacteria. If this is avoided, a therapy combining distinct antibiotics may be more successful in eradicating bacteria in tissues surrounding biomaterials^37^.

Due to the inherent instability of coatings deposited using spin-coating, the *Green* coating showed a reduction in its antibiofilm effect during longer incubation periods in 0.9% NaCl physiologic solution (Supplementary Fig. S11). This finding does not necessarily mean that the coating would not retain its antibiofilm effect if it was more firmly anchored to a substrate. The use of these coated materials for transient or short-term exposure is appropriate for spin-coated films; however, covalent attachment of the proanthocyanidins to the surface would most likely extend the lifetime of the coating and be necessary for long-term efficacy. With a covalent attachment it can be hypothesized that the coating would be as resilient on a materials surface as it was on bacterial surfaces (Fig. 3c), however this should be studied, and is a topic for further investigations.

**Figure 6:**
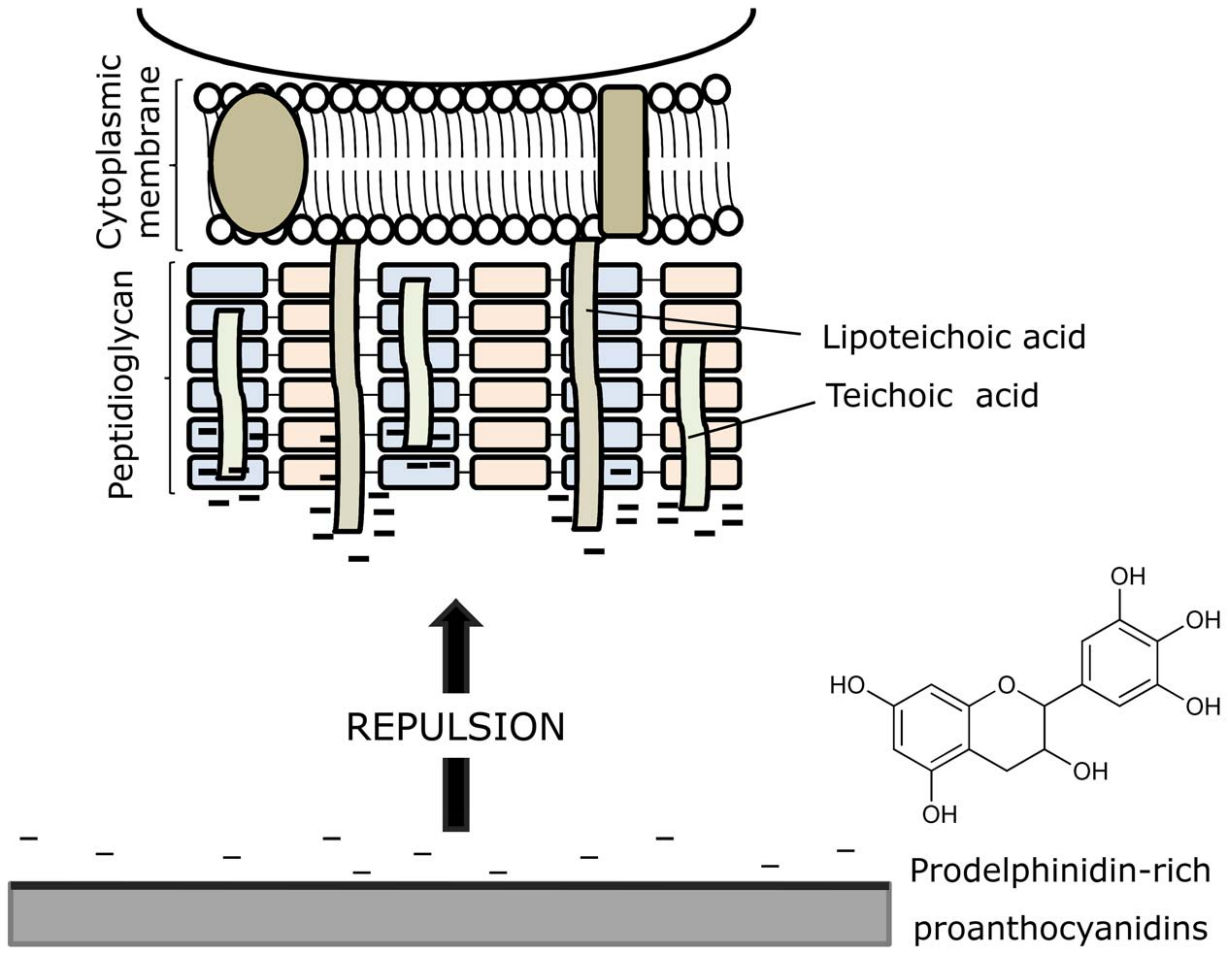
Mechanism proposed for inhibition of bacterial adhesion and biofilm formation by PMP-coated surfaces. It is based on repulsive forces between the anionic *S. epidermidis* surface and the negatively charged material surface after coating with proanthocyanidins.

The fact that bacterial adhesion is suppressed on surfaces that are *Green*-coated can be explained by a combination of factors: (i) electrostatic repulsion arises between the negatively charged Gram-positive bacterial surface and the negatively charged proanthocyanidin-coated surface (Fig. 6); (ii) high hydrophilic surface character is obtained on the coated material, which would reduce bacterial adhesion; and, (iii) the film produced by PMP coating possibly gives rise to steric hindrance that blocks bacterium-substratum interactions.

Effective control of biointerfacial interactions is the key to developing improved surfaces, including those related to infection-resistant medical materials^38^. Here we show that an anti-infective natural *Green*-coating is a promising candidate for surface films that combat adhesion of clinically important bacteria while they are still compatible with mammalian cells. Taking into account the huge biodiversity, this work has the potential to lead to the development of innovative preventive strategies that can result in lower costs in medical procedures associated to the indwelling devices.

## Methods

### Plant material

*Pityrocarpa moniliformis* leaves were collected at a national park, Parque Nacional do Catimbau (PARNA do Catimbau, Pernambuco, Brazil), under the authorization of the responsible authority Instituto Chico Mendes de Conservacão da Biodiversidade (ICMBio) (license SISBIO 16.806). Taxonomic identification was confirmed at the herbarium of Instituto Agronômico de Pernambuco (voucher IPA 84048).

### Extraction and purification of proanthocyanidins

The dried leaves of *P. moniliformis* were powdered and submitted to extraction with water by maceration for 24 h. The bioguided fractionation was used to investigate the active compounds present in the crude aqueous extract (129 g). The aqueous extract (15 g) was suspended in methanol (75 mL) providing two samples: a soluble fraction, inactive in the biological assay, and a bioactive and insoluble fraction (12.9 g). This methanol insoluble fraction was dried and further extracted with 70% aqueous acetone (3 × 60 mL). The active acetone soluble fraction was dried to give a brown powder (8.8 g), which was subjected to column chromatography through Sephadex^™^ LH-20^39^ (Sigma-Aldrich Co., St. Loius, USA) by applying 500 mg and eluting with 50 mL for each solvent (H_2_O, H_2_O:MeOH (7:3, 1:1 v/v), MeOH, H_2_O:(CH_3_)_2_CO (9:1, 7:3, 1:1, 3:7 v/v), (CH_3_)_2_CO) to obtain the fractions F1 (4360 mg), F2 (223 mg), F3 (314 mg), F4 (600 mg), F5 (167 mg), F6 (51 mg), F7 (329 mg), F8 (159 mg) and F9 (25 mg).

### MALDI-MS analyses

2,5-dihydroxibenzoic acid (DHB) was used as matrix to analyze the *P. moniliformis* fractions using UltrafleXtreme MALDI-TOF/TOF equipment (BrukerDaltonics, Bremen, Germany). The samples (2 mg) were dissolved in 200 μL of acetonitrile (ACN): water (30:70, v/v). The DHB matrix (20 mg mL^−1^) in ACN:H_2_O (30:70, v/v) with 0.1% trifluoroacetic acid was supplemented with 0.1 M of NaCl solution. The sample solution and the matrix were mixed in equal amounts and, spotted onto a ground stainless steel MALDI target (1 μL). The ratio of sample:matrix:cationizant agent was 1:1:0.1 (v/v/v). The compounds were identified by MS data and by a fragmentation pathway. The external calibration was conducted with a mixture of peptides (the peptide calibration standard II of Bruker). For MS analyses, the experimental conditions used were: pulsed ion extraction of 100 ns, laser frequency of 1000 Hz, reflectron mode, positive ion mode and 600 shots were averaged to record a mass spectrum. The ions selected were accelerated to 19 kV in the LIFT cell for MS/MS analyses.

### Surfaces, bacterial strain and culture conditions

Hydrophobic (polystyrene and Permanox^™^) and hydrophilic (glass) material models were used in the assays. Permanox slides were purchased from Nalge Nunc International and sterile 96-well polystyrene flat-bottom microtiter plates (Costar 3599) were purchased from Corning Inc. (USA). *Staphylococcus epidermidis* ATCC 35984, *S. aureus* ATCC 25904, *E. faecalis* ATCC 29212, *P. aeruginosa* ATCC 27853 and *K. pneumoniae* ATCC 700603 were grown in Mueller Hinton (MH) agar (Oxoid Ltd., England) and a bacterial suspension in sterile 0.9% NaCl, corresponding to optical density at 600 nm (OD_600_) of 0.150 (3 × 10^8^ CFU mL^−1^), was used in the assays.

### Adhesion and biofilm formation assay

Bacterial adhesion and biofilm formation was evaluated using the crystal violet assay^40^. For *S. epidermidis*, *P. aeruginosa* and *K. pneumonia* the culture medium was tryptone soya broth (TSB, Oxoid Ltd, England); for *E. faecalis*, TSB was supplemented with 1% glucose; and for *S. aureus*, the medium used was brain heart infusion broth (BHI, Oxoid Ltd, England). Aqueous solutions of *P. moniliformis* fractions were prepared using ultrapure MilliQ water (Millipore, Bedford, USA) and were sterilized by 0.22 μm filtration. The biofilm formation water control was considered to represent 100% of biofilm formation.

### *Green*-coated surfaces

Precisely 300 μL of a 4.0 mg mL^−1^ PMP solution in 70% aqueous acetone (Merck, Germany) was spin coated onto a Permanox slide (30 × 25 mm^2^) during a cycle of 500 rpm (5 s) and then accelerated to 5000 rpm (40 s) in the spin coater Laurell Model WS-650MZ-23NPP/LITE. After coating, the specimens were heat-treated (2 h at 80°C) to allow for film annealing and to remove any excess solvent. Samples were sterilized with UV light during 20 minutes and then were cut to produce 10 × 25 mm^2^ coated-substrates. As controls, samples spin coated with 300 μL of 70% aqueous acetone solution but without PMP and samples without coating were heated to 80°C and UV-treated.

### Surface characterization

Permanox samples were characterized before and after coating with PMP using X-ray photoelectron spectroscopy (XPS) and water contact angle (WCA) measurements and ellipsometry. For XPS, the chemical speciation of the C 1 s signal was achieved with an Omicron SPHERA spectrometer at a pass energy of 5 eV using Mg K*α* radiation (1253.6 eV). Spectral fitting was performed using CASA XPS software. Contact angle measurements were carried out using the sessile drop technique and double deionized water. The drop was observed directly using an Olympus BX-41 microscope objective lens and images were digitally captured using a 1.4 megapixel CCD camera. The reported water contact angles are means of more than five measurements performed on different areas of each sample surface.

Analysis of film thickness was done using a J.A. Woollam alpha-SE spectroscopic ellipsometer at 70° incidence angle in Long Mode (High-precision mode, data acquisition rate of 30 s). The samples were measured dry and with translucent adhesive tape applied to the back side of the slide to suppress backside reflection. The transparent substrate was determined through measurement of uncoated Permanox slides at three points and modeling the substrate optical properties using a B-spline. For each point, a model was subsequently constructed with an over layer described with a Cauchy film. Four randomly chosen measurement points were measured on two coated slides. The thicknesses were modeled for each point using all three models and the result from the model with the best fit was used for determining the average thickness. Thickness is given as an average of eight measurement points ± standard deviation. As a control, thicknesses were also modeled for acetone-treated Permanox slides.

### Cell culture

Mammalian epithelial Vero cell line was grown in Dulbecco's Modified Eagle Medium (DMEM) supplemented with 10% heat inactivated fetal bovine serum (FBS) and 1% penicillin/streptomycin and was incubated in an atmosphere of 5% CO_2_ at 37°C.

### Evaluation of PMP cytotoxicity

The cytotoxicity of PMP in solution was investigated using the MTT (Thiazolyl Blue Tetrazolium Bromide, Sigma-Aldrich, USA) assay^41^ and exponentially growing Vero cells at 1.5 × 10^4^ cells per well, in 96 well microplates. For “non-treated” cells, the PMP was replaced with water (100% of viability), and for the positive control, with 1% triton X-100 solution.

### Mammalian cells compatibility of *Green*-coated surfaces

Segments of the non-coated, acetone-treated, and PMP-coated Permanox were placed in the wells of 24-well tissue culture plates. Vero cells were seeded at 5 × 10^4^ cells per well. After incubation (24 h, 5% CO_2_ at 37°C), the cells that had grown on the coated Permanox were mildly fixed with 0.2% glutaraldehyde in PEM buffer (100 mM pipes, 1 mM EGTA, 2 mM MgCl_2_, pH 6.8) and subsequently in 2 mg mL^−1^ NaBH_4_ to reduce glutaraldehyde autofluorescence. The adherent cell microtubules were labelled using 1 μM fluorescent taxoid FLUTAX-2^42^ which was kindly provided by Dr. André A. Souto (Faculdade de Química, PUCRS, Brazil), and the DNA was stained with 10 μg mL^−1^ of DAPI (Sigma-Aldrich Co., USA). Images were obtained using an Olympus IX81 confocal microscope and UPLSAPO 60X W NA:1.20 objective and were overlaid using Olympus FV 1000 software.

### The race for the surface under static conditions

This experiment was conducted according Subbiahdoss and co-workers^26^, with some modifications. *S. epidermidis* was inoculated in 10 mL of TSB and cultured for 24 h at 37°C. Bacteria were harvested by centrifugation, washed twice with sterile water and further with sterile PBS. Subsequently, bacteria were sonicated on ice to disrupt clusters and a suspension of 3 × 10^8^ CFU mL^−1^ was prepared. Using the modified DMEM [98% (DMEM + FBS − without antibiotics) and 2% TSB], a suspension equivalent to 3 × 10^6^ CFU mL^−1^ was obtained.

The segments (1.0 × 1.0 cm^2^) of the non-coated, acetone-treated, and PMP-coated Permanox were placed in the wells of 24-well tissue culture plates and exposed to 500 μL of bacterial suspension during 30 min at 37°C. After, the segments were gently washed with PBS and transferred to another 24-well plate, where the Vero cells, suspended in the modified DMEM, were seeded at 5 × 10^4^ cells per well and maintained at 37°C, 5% CO_2_. In order to dilute toxic metabolites from bacteria, after 6 h of incubation, fresh modified DMEM was added to each well. Following 24 h incubation, samples were prepared as described in the section above and imaged by CLSM. The area occupied by *S. epidermidis* and Vero cells on on-coated, acetone-treated, and PMP-coated surfaces was measured in NIS Elements 3.2 software (Nikon). Measurements were done in 3 images for every type of surface. Only cells or bacteria clusters associated with surface were measured. In case of single bacterial cells, an average area taken by a bacterial cell was measured (n = 3), the total number of attached cells counted for every image was multiplied by the average area of a single cell.

### Statistical analyses

The data were expressed as the mean or the percent mean ± standard deviation (SD). Bacterial viability results were presented as mean ± SD of the log CFU mL^−1^. Each experiment was repeated three times. Statistical differences were determined using two-tailed Student's *t*-test and *p* value ≤0.05 was considered statistically significant.

## Author Contributions

D.S.T., R.B.G. and A.J.M. conceived and designed the research; M.V.S. was responsible for the collection and identification of vegetal material; D.S.T. performed the extraction and fractionation of proanthocyanidins and all biological experiments; D.B.S. and N.P.L. conducted chemical analysis of natural products. D.S.T. performed the scanning confocal microscopy, with a research assistant, under the supervision of E.L.P. and G.J. D.S.T. and A.P.F. carried out the experiments involving mammalian cells under the supervision of T.T. G.V.S. performed the X.P.S. and W.C.A. experiments to evaluate the coated surfaces and analyzed these data. O.R. and M.R. performed the ellipsometry experiments and the quantitative analysis of co-culture assay. D.S.T., D.B.S., R.B.G. and A.J.M. wrote the manuscript and the other authors revised the paper critically for important intellectual content.

## Supplementary Material

Supplementary Information Supplementary Information
