# Supplementary material for: Natural Green Coating Inhibits Adhesion of Clinically Important Bacteria
Source: Sci Rep. 2015 Feb 6;5:8287. doi: 10.1038/srep08287 (PMC4319173; doi:10.1038/srep08287)
Supplement: Supplementary Information [file srep08287-s1.pdf]

Supplementary Information

Natural *Green* Coating Inhibits Adhesion of Clinically Important Bacteria

Danielle S. Trentin[1,2], Denise B. Silva[3], Amanda P. Frasson[1], Olena Rzhepishevska[4], Márcia V. da Silva[5], Elinor de L. Pulcini[6], Garth James[6], Gabriel V. Soares[7], Tiana Tasca[1], Madeleine Ramstedt[4], Raquel B. Giordani[8], Norberto P. Lopes[3,*], and Alexandre J. Macedo[1,2,*]

[1]Faculdade de Farmácia, Universidade Federal do Rio Grande do Sul, Porto Alegre/RS, 90610-000, Brazil.

[2]Centro de Biotecnologia, Universidade Federal do Rio Grande do Sul, Porto Alegre/RS, 91501-970, Brazil.

[3]Núcleo de Pesquisas em Produtos Naturais e Sintéticos (NPPNS), Faculdade de Ciências Farmacêuticas de Ribeirão Preto, Universidade de São Paulo, Ribeirão Preto/SP, 14040-903, Brazil.

[4]Department of Chemistry, Umeå University, Umeå, SE-90187, Sweden.

[5]Centro de Ciências Biológicas and Departamento de Bioquímica, Universidade Federal de Pernambuco, 50670-901 Recife, Brazil.

[6]Center for Biofilm Engineering, Montana State University, Bozeman/MT, 59717, United States of America.

[7]Instituto de Física, Universidade Federal do Rio Grande do Sul, Porto Alegre/RS, 91509-900, Brazil.

[8]Centro de Ciências da Saúde, Departamento de Farmácia, Universidade Federal do Rio Grande do Norte, Natal/RN, 59010-180, Brazil.

***Corresponding authors:**
Dr. Norberto P. Lopes
Núcleo de Pesquisa em Produtos Naturais e Sintéticos (NPPNS)
Faculdade de Ciências Farmacêuticas de Ribeirão Preto
Universidade de São Paulo, Ribeirão Preto/SP, Brazil.
E-mail: npelopes@fcfrp.usp.br;

Dr. Alexandre J. Macedo
Faculdade de Farmácia and Centro de Biotecnologia
Universidade Federal do Rio Grande do Sul, Porto Alegre/RS, Brazil.
E-mail: alexandre.macedo@ufrgs.br.

Table of contents **Page**

1. Data 1: Effect of *P. moniliformis* fractions on *S. epidermidis* biofilm formation and growth **4**

2. Chemical analyses of the fractions **5**

Data and Discussion 1: MALDI-MS: Identification of proanthocyanidins from the *P. moniliformis* fractions **5**

Data and Discussion 2: LC-DAD-MS - Identification of flavonoids from the fractions by UV, MS (ESI ionization) and MS/MS data **13**

3. Data and Discussion 3: Involvement of iron on *Staphylococcus* biofilm formation **18**

4. Data 2: Assessment viability of planktonic cells and water resistance of the coating **20**

5. Methods **21**

5.1. UPLC-DAD-MS analyses **21**

5.2 Pre-treatment of bacterial cells with PMP prior to adhesion and viability assays **21**

5.3 Bacterial growth and viability **21**

5.4 Scanning Electron Microscopy **22**

5.5 Confocal Laser Scanning Microscopy **22**

5.6 Iron chelating assessment **22**

5.7 Bacterial surface hydrophobicity index **23**

5.8 Non-biological model particle adhesion experiments **23**

5.9 Water resistance of the coating **23**

6. References **24**

**Tables and Figures**	**Page**
**Table S1** Distribution of polyflavan-3-ol oligomers and polymers analyzed by MALDI-TOF for proanthocyanidins from *P. moniliformis* leaves.	**7**
**Table S2** MS/MS data obtained by MALDI-LIFT (positive ion mode) from *P. moniliformis* proanthocyanidins.	**11**
**Table S3** Identification of chromatographic peaks and their UV, MS and MS/MS data.	**16**
**Figure S1.** Effect of *P. moniliformis* fractions obtained from Sephadex LH-20 on (a) *S. epidermidis* biofilm formation and on (b) *S. epidermidis* growth. * represents statistical difference in relation to the non-treated samples.	**4**
**Figure S2** MALDI-MS spectra (positive ionization mode) of the fractions F1, F2, F3, F4, F5, F6, F8 and F9. * represents the ions of matrix. The spectrum of F7 (PMP) is illustrated in Figure 1 (manuscript).	**10**
**Figure S3** MALDI-MS/MS spectra of the ions at (a) *m/z* 617 and (b) 1225.	**12**
**Figure S4** MALDI-MS/MS spectra of the ions at (a) *m/z* 633 and (b) 1241.	**12**
**Figure S5** MALDI-MS/MS spectra of the ions at (a) *m/z* 785 and (b) 1393.	**12**
**Figure S6.** MALDI-MS/MS spectra of the ions at (a) *m/z* 889 and (b) 1801.	**13**
**Figure S7** MALDI-MS/MS spectra of the ions at (a) *m/z* 1345 and (b) 2257.	**13**
**Figure S8** The aglycones of flavonoids identified in glycosides from *P. moniliformis* leaves: (a) quercetagetin, (b) myricetin and (c) apigenin.	**14**
**Figure S9** LC-DAD profile of the fractions F1-F9.	**15**
**Figure S10** Iron-chelating assessment.	**19**
**Figure S11** Assessment viability of planktonic cells and water resistance of the coating	**20**

1. Data 1: Effect of *P. moniliformis* fractions on *S. epidermidis* biofilm formation and growth

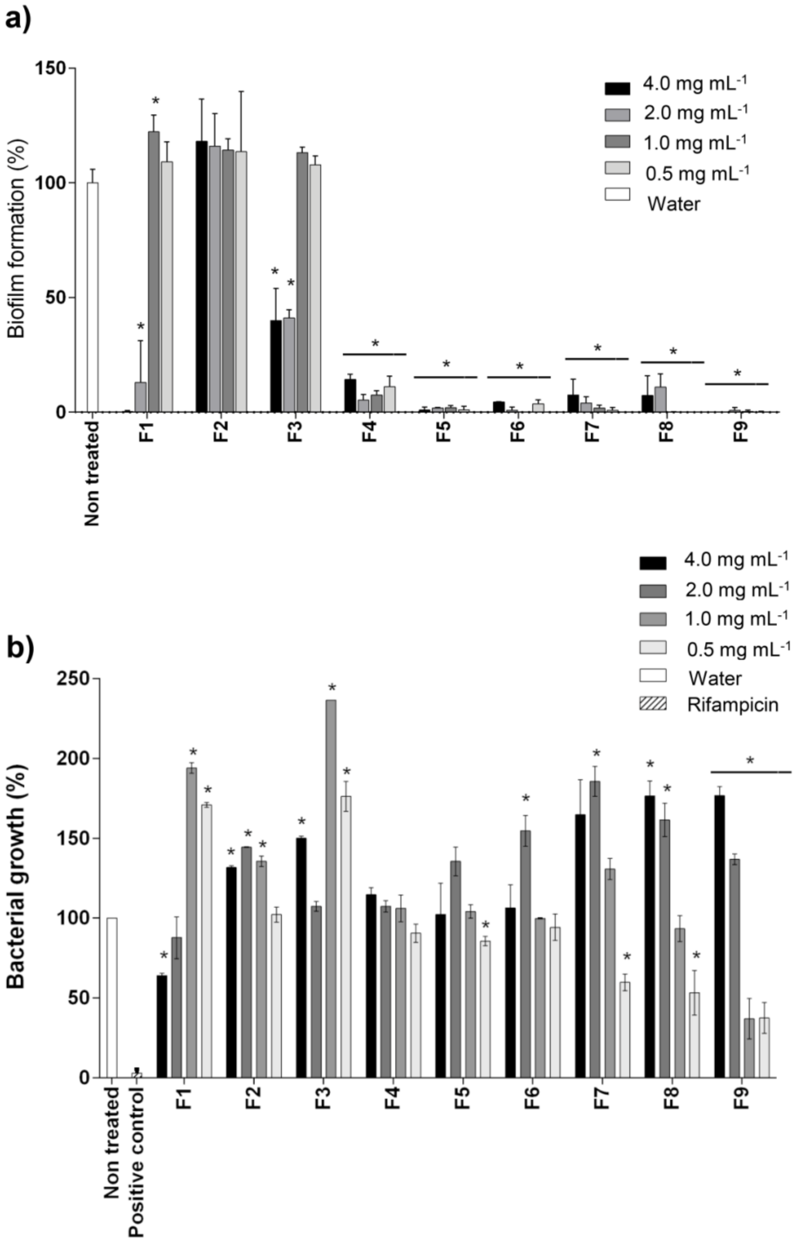

Figure S1 Effect of *P. moniliformis* fractions obtained from Sephadex LH-20 on (a) *S. epidermidis* biofilm formation and on (b) *S. epidermidis* growth. * represents statistical difference in relation to the non-treated samples.

2. Chemical analyses of the fractions

Supplementary Data and Discussion 1: MALDI-MS: Identification of proanthocyanidins from the *P. moniliformis* fractions

MALDI-MS and MS/MS analyses allowed the determination of polymer chain length, chemical constitution of individual polymer, as well as the sequential monomer units which produce each oligomers, allowing us to propose the chemical characterization of proanthocyanidins with variable degree of polymerization. The fractions F1 to F3 did not show tannins in your compositions (Supplementary Fig. S2), but five different polymeric series, distributed from F4 to F9 fractions, were identified in our study (Fig. 1, Supplementary Fig. S2-S7; Supplementary Table S1 and S2) and were described as follow:

Polymeric series from ion *m/z* 889 = 1PGU – nPDE (Supplementary Fig. S2 and S6; Supplementary Table S1 and S2). The polymeric series that started with ion *m/z* 889 was detected in fractions F4, F5, F8 and F9, and are formed by being composed up to 12 monomer units in the last two fractions. The MS/MS of *m/z* 889 $[M+Na]^+$ yielded the fragment ions *m/z* 721, 703, 583, 565, 417, 279 (Supplementary Table S2 and Supplementary Fig. S6), where the fragment ions *m/z* 583 and 279 represent the loss of prodelphinidin units, and the fragment ions *m/z* 279 corresponds to the presence of a proguibourtinidin unit. The fragment ions *m/z* 721 and 417 were produced by Retro Diels-Alder (RDA) fission of ring C (loss of 168 *u*) and confirmed the ring B substituents of the units proposed (Fig. 1b). Subsequently, these ions lost a H_2O molecule, which originates from the hydroxyl group at C-3. In MS/MS spectrum of octamer (*m/z* 2409), relevant fragments ions were produced as *m/z* 2105, 1801, 1495, 1191, 887, 583 and 279, which correspond to the consecutive loss of one, two and up to seven prodelphinidin units (Supplementary Table S2 and Supplementary Fig. S6).

Polymeric series from ion *m/z* 633 =nPDE (Supplementary Fig. S2 and S4; Supplementary Table S1 and S2). The polymeric series of the ions *m/z* 633, 937, 1241, 1545, 1849, 2153, 2457, 2761 and 3065 is composed only by prodelphinidin units with B-type linkages

and it was observed in fractions F5, F6, F7 and F8. The dimer *m/z* 633 $[M+Na]^+$ yielded the fragment ions *m/z* 495, 465, 447, 371, 327, 297, 279 and 161 and was observed only from F5 and F6. The RDA fission led to the fragments *m/z* 495 and 161, confirming the hydroxyl substituents in the B ring of prodelphinidin units (Supplementary Table S2 and Supplementary Fig. S4). Moreover, the fragment ion *m/z* 327 is relative to the consecutive loss of one prodelphinidin unit, confirming the coupling between two prodelphinidin units.

Polymeric series from ion *m/z* 617 = 1 PRO/PCY – nPDE (Supplementary Fig. S2 and S3; Supplementary Table S1 and S2). Regarding the series of the ions *m/z* 617, 921, 1225, 1529, 1833, 2137, 2441 and 2745, it is composed by two kinds of oligomers: one has a prorobinetidin, the other has a procyanidin, and both present prodelphinidin units added consecutively. The presence of procyanidin and prorobinetidin in these series was confirmed by the loss of 152 and 168 *u*, which correspond to two and three hydroxyl groups in the ring B (Supplementary Table S2 and Supplementary Fig. S3). This series was identified in the fractions F6 and F7.

Polymeric series from ion *m/z* 1345 = 1PGU-1GAL – nPDE (Supplementary Fig. S2 and S7; Supplementary Table S1 and S2) and from ion *m/z* 1089 = 2PDE-1GAL – nPDE (Supplementary Fig. S2 and S5; Supplementary Table S1 and S2). The other two polymeric series (*m/z* 1345, 1649, 1953, 2257, 2561, 2865, 3169, 3473 and *m/z*785, 1089, 1393, 1697, 2001, 2305, 2609) were identified in fractions F8 and F9, and F6 and F7, respectively, and they showed differences of 152 *u* when compared with the series from *m/z* 889 and 633, respectively. In the MS/MS spectra of these oligomers, the losses of 152 *u* were observed and they are related to galloyl group. Moreover, losses of 168 *u* were also observed and they confirmed prodelphinidin units (Supplementary Table S2; Supplementary Fig. S5 and S7).

Table S1. Distribution of polyflavan-3-ol oligomers and polymers analyzed by MALDI-TOF for proanthocyanidins from *P. moniliformis* leaves.

Fraction		$[M+Na]^+$	MF	Compound**
**F1**		-	-	-
**F2***		-	-	-
**F3***		-	-	-
**F4***	Series A	889	$C_{45}H_{38}O_{18}$	1 PGU – 2 PDE
		1193	$C_{60}H_{50}O_{25}$	1 PGU – 3 PDE
		1497	$C_{75}H_{62}O_{32}$	1 PGU – 4 PDE
		1801	$C_{90}H_{74}O_{39}$	1 PGU – 5 PDE
		2105	$C_{105}H_{86}O_{46}$	1 PGU – 6 PDE
		2409	$C_{120}H_{98}O_{53}$	1 PGU – 7 PDE
		2713	$C_{135}H_{110}O_{60}$	1 PGU – 8 PDE
		3017	$C_{150}H_{122}O_{67}$	1 PGU – 9 PDE
**F5***	Series A	889	$C_{45}H_{38}O_{18}$	1 PGU – 2 PDE
		1193	$C_{60}H_{50}O_{25}$	1 PGU – 3 PDE
		1497	$C_{75}H_{62}O_{32}$	1 PGU – 4 PDE
		1801	$C_{90}H_{74}O_{39}$	1 PGU – 5 PDE
		2105	$C_{105}H_{86}O_{46}$	1 PGU – 6 PDE
		2409	$C_{120}H_{98}O_{53}$	1 PGU – 7 PDE
		2713	$C_{135}H_{110}O_{60}$	1 PGU – 8 PDE
		3017	$C_{150}H_{122}O_{67}$	1 PGU – 9 PDE
		3321	$C_{165}H_{134}O_{74}$	1 PGU – 10 PDE
	Series B	633	$C_{30}H_{26}O_{14}$	2 PDE
		937	$C_{45}H_{38}O_{21}$	3 PDE
		1241	$C_{60}H_{50}O_{28}$	4 PDE
		1545	$C_{75}H_{62}O_{35}$	5 PDE
		1849	$C_{90}H_{74}O_{42}$	6 PDE
		2153	$C_{105}H_{86}O_{49}$	7 PDE
		2457	$C_{120}H_{98}O_{56}$	8 PDE
**F6**	Series A	633	$C_{30}H_{26}O_{14}$	2 PDE
		937	$C_{45}H_{38}O_{21}$	3 PDE
		1241	$C_{60}H_{50}O_{28}$	4 PDE
		1545	$C_{75}H_{62}O_{35}$	5 PDE
		1849	$C_{90}H_{74}O_{42}$	6 PDE
		2153	$C_{105}H_{86}O_{49}$	7 PDE
		2457	$C_{120}H_{98}O_{56}$	8 PDE
	Series B	617	$C_{30}H_{26}O_{13}$	1 PDE – 1 PRO/PCY
		921	$C_{45}H_{38}O_{20}$	1 PDE – 1 PRO/PCY – PDE
		1225	$C_{60}H_{50}O_{27}$	1 PDE – 1 PRO/PCY – 2 PDE
		1529	$C_{75}H_{62}O_{34}$	1 PDE – 1 PRO/PCY – 3 PDE
		1833	$C_{90}H_{74}O_{41}$	1 PDE – 1 PRO/PCY – 4 PDE
		2137	$C_{105}H_{86}O_{48}$	1 PDE – 1 PRO/PCY – 5 PDE
		2441	$C_{120}H_{98}O_{55}$	1 PDE – 1 PRO/PCY – 6 PDE
	Series C	785	$C_{37}H_{30}O_{18}$	2 PDE – 1 GAL
		1089	$C_{52}H_{42}O_{25}$	2 PDE – 1 GAL – 1 PDE
		1393	$C_{67}H_{54}O_{32}$	2 PDE – 1 GAL – 2 PDE
		1697	$C_{82}H_{66}O_{39}$	2 PDE – 1 GAL – 3 PDE
		2001	$C_{97}H_{78}O_{46}$	2 PDE – 1 GAL – 4 PDE
		2305	$C_{112}H_{90}O_{53}$	2 PDE – 1 GAL – 5 PDE (cont.)

**F7 - PMP**	Series A	937	$C_{45}H_{38}O_{21}$	3 PDE
		1241	$C_{60}H_{50}O_{28}$	4 PDE
		1545	$C_{75}H_{62}O_{35}$	5 PDE
		1849	$C_{90}H_{74}O_{42}$	6 PDE
		2153	$C_{105}H_{86}O_{49}$	7 PDE
		2457	$C_{120}H_{98}O_{56}$	8 PDE
		2761	$C_{135}H_{110}O_{63}$	9 PDE
		3065	$C_{150}H_{122}O_{70}$	10 PDE
	Series B	921	$C_{45}H_{38}O_{20}$	1 PDE – 1 PRO/PCY – 1 PDE
		1225	$C_{60}H_{50}O_{27}$	1 PDE – 1 PRO/ PCY – 2 PDE
		1529	$C_{75}H_{62}O_{34}$	1 PDE – 1 PRO/PCY – 3 PDE
		1833	$C_{90}H_{74}O_{41}$	1 PDE – 1 PRO/PCY – 4 PDE
		2137	$C_{105}H_{86}O_{48}$	1 PDE – 1 PRO/PCY – 5 PDE
		2441	$C_{120}H_{98}O_{55}$	1 PDE – 1 PRO/PCY – 6 PDE
		2745	$C_{135}H_{110}O_{62}$	1 PDE – 1 PRO/PCY – 7 PDE
	Series C	1089	$C_{52}H_{42}O_{25}$	2 PDE – 1 GAL - 1 PDE
		1393	$C_{67}H_{54}O_{32}$	2 PDE – 1 GAL – 2 PDE
		1697	$C_{82}H_{66}O_{39}$	2 PDE – 1 GAL – 3 PDE
		2001	$C_{97}H_{78}O_{46}$	2 PDE – 1 GAL – 4 PDE
		2305	$C_{112}H_{90}O_{53}$	2 PDE – 1 GAL – 5 PDE
		2609	$C_{127}H_{102}O_{60}$	2 PDE – 1 GAL – 6 PDE
**F8**	Series A	889	$C_{45}H_{38}O_{18}$	1 PGU – 2 PDE
		1193	$C_{60}H_{50}O_{25}$	1 PGU – 3 PDE
		1497	$C_{75}H_{62}O_{32}$	1 PGU – 4 PDE
		1801	$C_{90}H_{74}O_{39}$	1 PGU – 5 PDE
		2105	$C_{105}H_{86}O_{46}$	1 PGU – 6 PDE
		2409	$C_{120}H_{98}O_{53}$	1 PGU – 7 PDE
		2713	$C_{135}H_{110}O_{60}$	1 PGU – 8 PDE
		3017	$C_{150}H_{122}O_{67}$	1 PGU – 9 PDE
		3321	$C_{165}H_{134}O_{74}$	1 PGU – 10 PDE
		3625	$C_{180}H_{146}O_{81}$	1 PGU – 11 PDE
	Series B	937	$C_{45}H_{38}O_{21}$	3 PDE
		1241	$C_{60}H_{50}O_{28}$	4 PDE
		1545	$C_{75}H_{62}O_{35}$	5 PDE
		1849	$C_{90}H_{74}O_{42}$	6 PDE
		2153	$C_{105}H_{86}O_{49}$	7 PDE
	Series C	1345	$C_{67}H_{54}O_{29}$	1 PGU – 1 GAL - 3 PDE
		1649	$C_{82}H_{66}O_{36}$	1 PGU – 1 GAL - 4 PDE
		1953	$C_{97}H_{78}O_{43}$	1 PGU – 1 GAL - 5 PDE
		2257	$C_{112}H_{90}O_{50}$	1 PGU – 1 GAL - 6 PDE
		2561	$C_{127}H_{102}O_{57}$	1 PGU – 1 GAL - 7 PDE
		2865	$C_{142}H_{114}O_{64}$	1 PGU – 1 GAL -8 PDE
		3169	$C_{157}H_{126}O_{71}$	1 PGU – 1 GAL - 9 PDE
		3473	$C_{172}H_{138}O_{78}$	1 PGU – 1 GAL - 10 PDE
**F9**	Series A	889	$C_{45}H_{38}O_{18}$	1 PGU – 2 PDE
		1193	$C_{60}H_{50}O_{25}$	1 PGU – 3 PDE
		1497	$C_{75}H_{62}O_{32}$	1 PGU – 4 PDE
		1801	$C_{90}H_{74}O_{39}$	1 PGU – 5 PDE
		2105	$C_{105}H_{86}O_{46}$	1 PGU – 6 PDE
		2409	$C_{120}H_{98}O_{53}$	1 PGU – 7 PDE
		2713	$C_{135}H_{110}O_{60}$	1 PGU – 8 PDE
		3017	$C_{150}H_{122}O_{67}$	1 PGU – 9 PDE
		3321	$C_{165}H_{134}O_{74}$	1 PGU – 10 PDE

(cont.)

	3625	$C_{180}H_{146}O_{81}$	1 PGU – 11 PDE
Series B	1345	$C_{67}H_{54}O_{29}$	1 PGU – 1 GAL - 3 PDE
	1649	$C_{82}H_{66}O_{36}$	1 PGU – 1 GAL - 4 PDE
	1953	$C_{97}H_{78}O_{43}$	1 PGU – 1 GAL - 5 PDE
	2257	$C_{112}H_{90}O_{50}$	1 PGU – 1 GAL - 6 PDE
	2561	$C_{127}H_{102}O_{57}$	1 PGU – 1 GAL - 7 PDE
	2865	$C_{142}H_{114}O_{64}$	1 PGU – 1 GAL -8 PDE

*Flavonoids were identified in this fraction (see Supplementary Table S3); **Proanthocyanidins identified by MS and MS/MS data. MF: molecular formula, PGU: proguibourtinidin, PDE: prodelphinidin; PRO: prorobinetinidin; PCY: procyanidin, GAL: galloyl

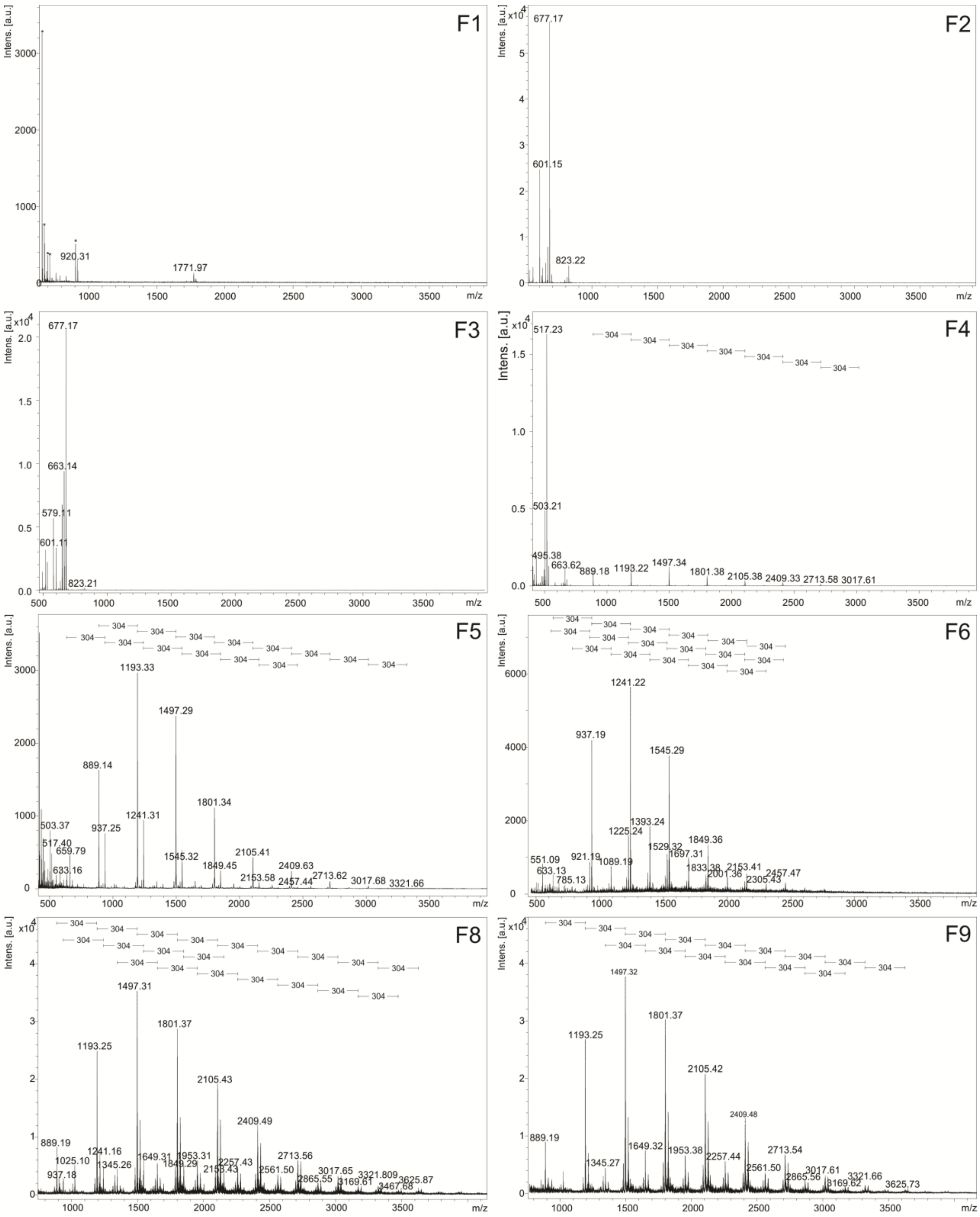

Figure S2 MALDI-MS spectra (positive ionization mode) of the fractions F1, F2, F3, F4, F5, F6, F8 and F9.* represents the ions of matrix. The spectrum of F7 (PMP) is illustrated in Fig. 1 (manuscript).

Table S2. MS/MS data obtained by MALDI-LIFT (positive ion mode) from *P. moniliformis* proanthocyanidins.

$[M+Na]^+$ (*m/z*)	MS/MS (*m/z*)	$[M+Na]^+$ (*m/z*)	MS/MS (*m/z*)
**617**	597, 465, 449, 447, 429, 327, 311	**1545**	1377, 1359, 1241, 1239, 937, 935, 305, 1055, 769, 749, 633, 631, 465, 327, 287
**633**	495, 465, 447, 371, 327, 297,279, 161	**1649**	1481, 1345, 1193, 1039, 887, 583, 565, 287, 279
**785**	631, 479, 287	**1697**	1529, 1511, 1393, 1241, 1087, 935, 783, 631, 441, 287
**889**	721, 703, 583, 565, 541, 417, 405, 317, 299, 287, 279, 179, 139	**1801**	1633, 1615, 1495, 1191, 887, 869, 583, 565, 405, 279
**921**	753, 735, 631, 615, 447, 327, 309	**1849**	1681, 1663, 1545, 1543, 1359, 1241, 1239, 1222, 1053, 937, 935, 769, 633, 631, 465, 327, 287
**937**	769, 751, 633, 465, 327	**1953**	1785, 1649, 1343, 1193, 1039, 887, 701, 583, 565, 279, 179
**1089**	921, 903, 783, 785, 633, 479	**2001**	1849, 1833, 1815, 1697, 1545, 1391, 1241, 1087, 935, 783, 631, 479, 423, 287
**1193**	1025, 1007, 887, 701, 583, 565, 541, 417, 405, 317, 299, 279,179, 139	**2105**	1937, 1919, 1801, 1633, 1615, 1497, 1495, 1191, 1173, 1005, 887, 869, 701, 583, 565, 405, 279
**1225**	1057, 1039, 921, 631, 615, 447, 317, 305	**2153**	1967, 1849, 1543, 1241, 937, 935, 749, 633, 631
**1241**	1073, 1055, 937, 935, 769, 749, 633, 631, 465, 447	**2257**	2089, 2071, 1951, 1647, 1495, 1343, 1191, 1039, 887, 701, 583, 565, 279
**1345**	1177, 1159, 887, 583, 735, 565, 279, 179, 139	**2409**	2241, 2223, 2105, 1937, 1801, 1495, 1477, 1191, 1173, 1005, 887, 869, 701, 583, 565, 305, 287, 279, 179
**1393**	1225, 1207, 1087, 937, 783, 633, 479	**2457**	2422, 2273, 2153, 1849, 1545, 1241, 1239, 937, 935, 631, 583, 287
**1497**	1329, 1311, 1191, 887, 701, 583, 565 441, 423, 405, 305, 287, 279, 179	**2561**	1039, 887, 701, 583, 565, 279
**1529**	1361, 1343, 1225, 921, 919, 935, 609	**2713**	2409, 2103, 1801, 1495, 1191, 887, 583, 565, 405, 287, 279, 179

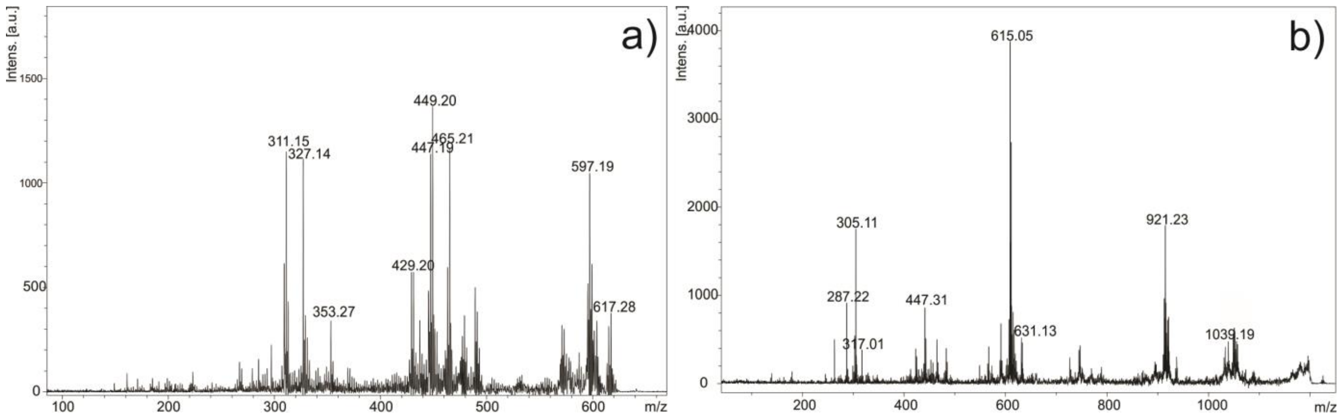

Figure S3 MALDI-MS/MS spectra of the ions at (a) *m/z* 617 and (b) 1225.

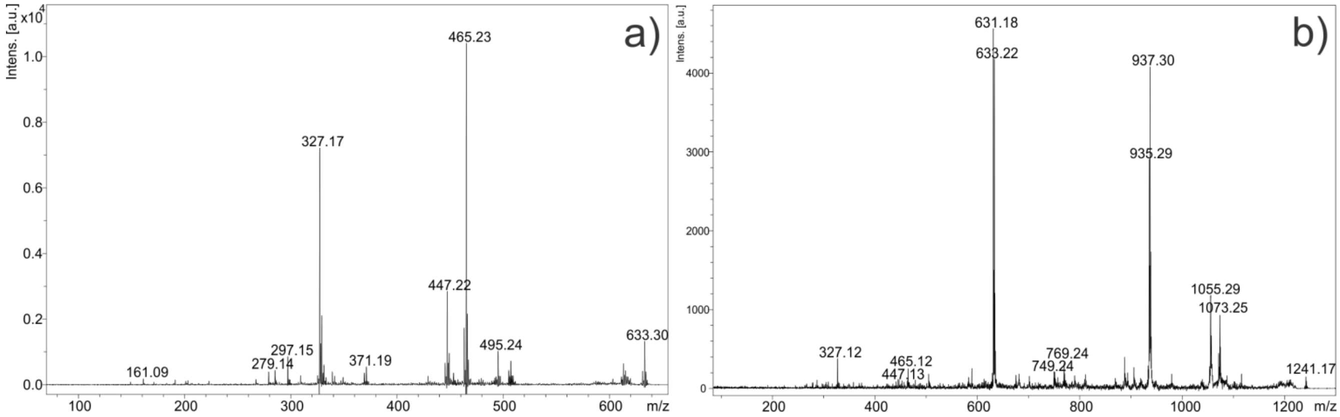

Figure S4 MALDI-MS/MS spectra of the ions at (a) *m/z* 633 and (b) 1241.

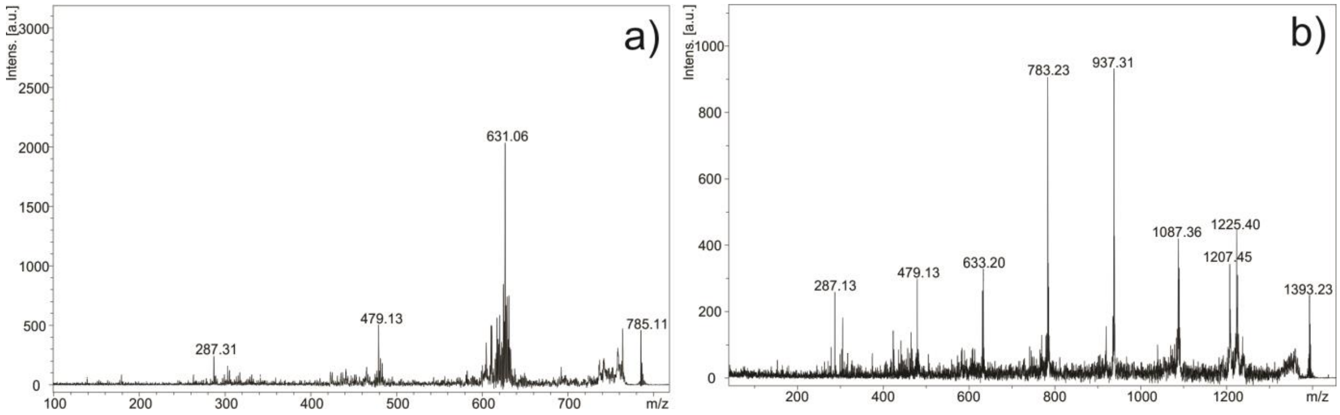

Figure S5 MALDI-MS/MS spectra of the ions at (a) *m/z* 785 and (b) 1393.

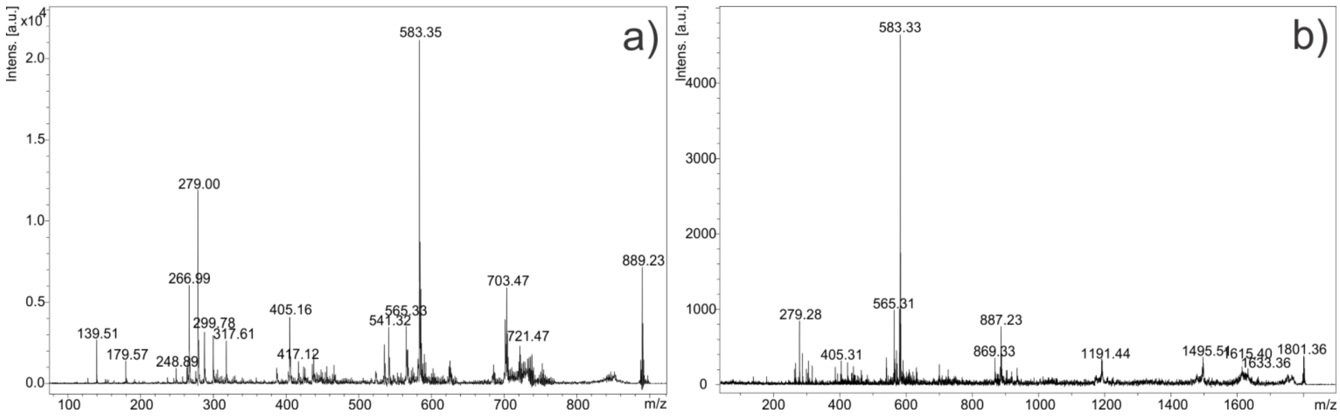

Figure S6 MALDI-MS/MS spectra of the ions at (a) *m/z* 889 and (b) 1801.

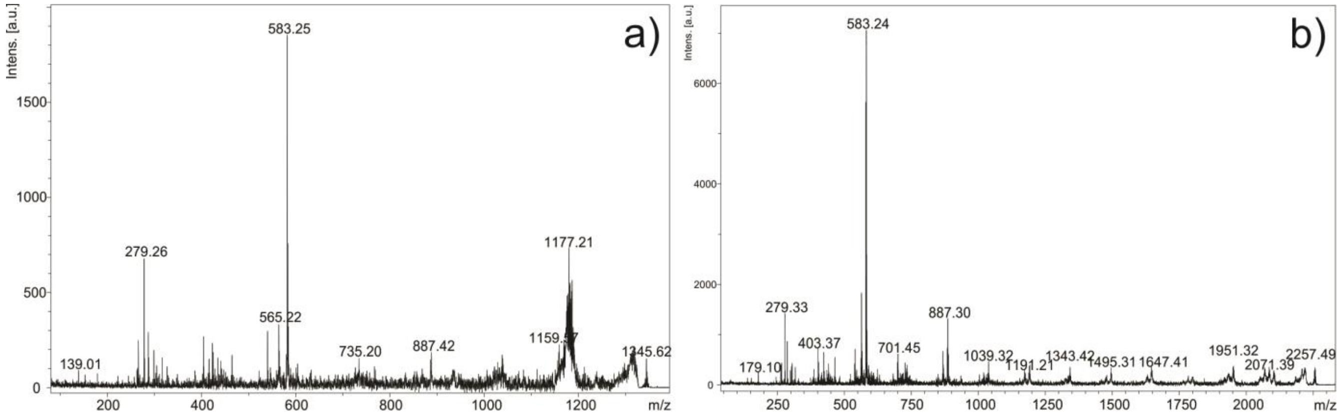

Figure S7 MALDI-MS/MS spectra of the ions at (a) *m/z* 1345 and (b) 2257.

Supplementary Data and Discussion 2: LC-DAD-MS - Identification of flavonoids from the fractions by UV, MS (ESI ionization) and MS/MS data.

The F6-F9 fractions did not show flavonoids in their composition (Supplementary Fig. S9). The fraction F1 did not present proanthocyanidins or other UV detectable compounds (Supplementary Fig.S2 and S9, respectively), so we suggest that it contains sugars.

The fraction F2 and F3 also did not show tannins in their composition (Supplementary Fig. S2). However, the UV data obtained for the compounds of fractions F2-F5 (Supplementary Fig. S9 and Supplementary Table S3) revealed that the substance **1** showed a UV spectrum characteristic of flavones ($\lambda_{max} \approx 260$ and 340 nm), while the other peaks showed spectra of flavonols[1]. Glycosylated flavonoids were identified in the fractions F2 - F4 and they are *O*-glycosylated. In all

MS/MS spectra, important product ions for the structural identification of the flavonoids were observed, which are produced from the specific A- and B-ring fragmentation pathway due to retro-Diels-Alder fragmentation reaction of C-ring, so they are diagnostic ions for the A- and B-ring substitution pattern[2,3]. The flavonoids **2, 3** and **5** revealed losses of 162 *u* and 146 *u* that correspond, respectively, to hexoside and deoxyhexoside, while **1** and **4** showed only losses of one sugar (162 *u*). Thus, apigenin *O*-hexosyl-deoxihexoside (**2**) and quercetagetin dimethyl-*O*-hexosyl-deoxihexoside (**5**) were identified in the fraction F2, apart from these the compound quercetagetin methyl-*O*-hexosyl-deoxihexoside (**3**) was identified in fraction F3. In the fraction F4, quercetagetin *O*-hexoside (**1**), quercetagetin methyl-*O*-hexoside (**4**) and myricetin methyl ether (**7**) were identified (Supplementary Table S3, Supplementary Fig. S8 and S9). Among the flavonoids, only flavonol aglycones (compounds **6** and **7**) were observed in the fraction F5, but this sample also has proanthocyanidins (Supplementary Table S1, Supplementary Fig. S2). For the compound **7**, an additional loss of 15 *u* (*m/z* 316, positive mode) was observed, confirming the presence of a methyl group. All MS/MS data of compounds **6** and **7** were compatible with myricetin and myricetin methyl ether, respectively (Supplementary Table S3).

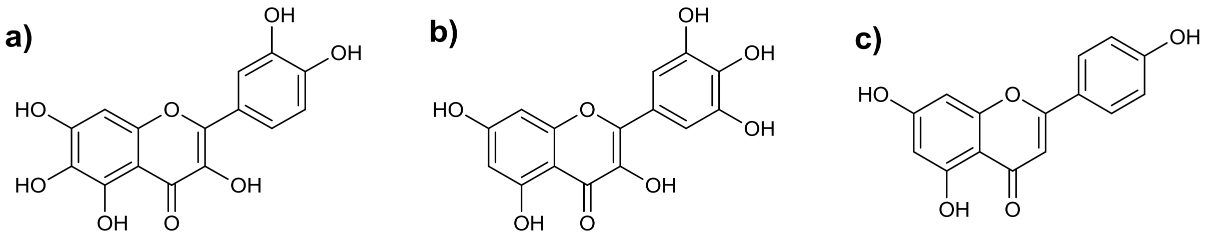

Figure S8 The aglycones of flavonoids identified in glycosides from *P. moniliformis* leaves: (a) quercetagetin, (b) myricetin and (c) apigenin.

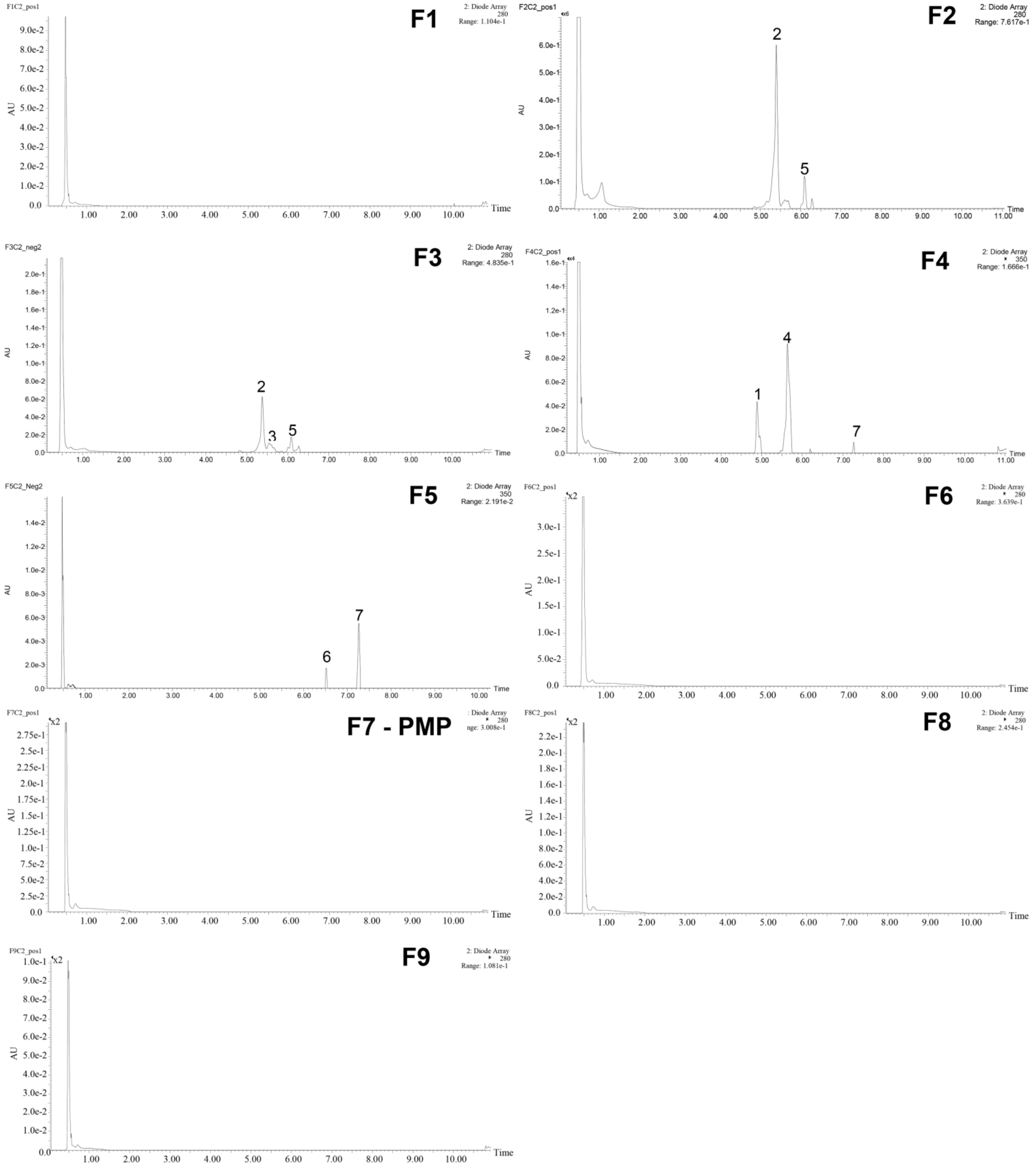

Figure S9 LC-DAD profile of the fractions F1-F9.

Table S3 Identification of chromatographic peaks and their UV, MS and MS/MS data.

Peak	Compound	RT (min)	UV max (nm)	Negative Ions		Positive Ions	
				MS (*m/z*)	MS/MS (*m/z*)	MS (*m/z*)	MS/MS (*m/z*)
1	Quercetagetin *O*-hexoside	4.89	265, 355	479 $[M-H]^-$	479→ 317[bp], 271, 247, 214, 179, 163	481 $[M+H]^+$	481→ 319[bp], 165, 146, 111, 97, 85<br>319→ 302, 256, 235, 218, 172, 165, 153[bp], 137, 109, 95
2	Apigenin *O*-hexosyl-deoxihexoside	5.40	269, 338	577 $[M-H]^-$	577→ 457, 413[bp], 323, 293	579 $[M+H]^+$	579→ 561, 433[bp], 416, 398, 367, 337, 313, 129<br>433→ 416, 397, 368, 349, 338, 314, 283[bp]
3	Quercetagetin methyl-*O*-hexosyl-deoxihexoside	5.55	265, 356	639 $[M-H]^-$	639→480, 374, 331[bp], 316, 301, 191, 179, 163, 151<br>577→415[bp]	641 $[M+H]^+$	641→ 495, 479, 333[bp], 163, 146<br>495→ 333[bp], 145, 127
4	Quercetagetin methyl-*O*-hexoside	5.61	265, 355	493 $[M-H]^-$	493→ 331[bp], 316, 300, 287, 269, 244, 199, 179, 179, 151	495 $[M+H]^+$<br>517 $[M+Na]^+$	495→ 333[bp], 318, 301, 274, 216, 165<br>333→ 319, 301, 289, 284, 245[bp], 234, 217, 208, 189, 153, 107
5	Quercetagetin dimethyl-*O*-hexosyl-deoxihexoside	6.09	255, 358	653 $[M-H]^-$	653→622, 358, 345[bp], 329, 315, 298, 277, 259, 151	655 $[M+H]^+$	655→347[bp], 164, 153, 147, 129<br>509→347[bp], 145<br>347→332[bp], 317, 291, 286, 261, 163, 153, 139
6	Myricetin	6.52	255, 375	317 $[M-H]^-$	317→ 271, 245, 203, 199, 179, 165, 151[bp], 135, 124, 107	319 $[M+H]^+$	319→256, 245, 221, 217, 207, 179, 167, 163, 153[bp], 137, 111
7	Myricetin methyl ether	7.26	265, 374	331 $[M-H]^-$	331→ 316[bp], 288, 269, 232, 216, 203, 192, 179, 164, 151, 136, 123	333 $[M+H]^+$	333→ 301, 290, 274[bp], 260, 227, 219, 206, 171, 167, 137

RT: retention time; bp: base peak (100% relative intensity).

In summary, our results show that (i) the fractions F2 and F3, composed by glycosylated flavonoids, were not active or prevented just 50% of *S. epidermidis* biofilm formation when high concentrations were used; (ii) F4 spectrum (MALDI-MS) showed the ions related to flavonoids with higher intensity than the ions of proanthocyanidins that suggested higher concentration of flavonoids, and this fraction confer an important, though not complete, antibiofilm action; (iii) the fractions F5-F9 were able to prevent biofilm formation completely, and they are composed by high amount of proanthocyanidins; and (iv) levels of flavonoids in active fraction F5 were lower than those of proanthocyanidins and fractions F6-F9 are flavonoid free. Taking together, these findings

allowed us to indicate that *P. moniliformis* proanthocyanidins are the compounds responsible for the inhibition of biofilm formation by *S. epidermidis*.

Additionally, to the best of our knowledge, the occurrence of proguibourtinidin (Fig. 1b) in the Fabaceae family is described for the first time. On the other hand, procyanidin and prodelphinidin (Fig. 1b) residues are widespread in this family. In this sense, the study of *Parapiptadenia rigida*, which is taxonomically related to *P. moniliformis*, also demonstrated the presence of dimeric prodelphinidin and procyanidin in the bark[4].

3. Supplementary Data and Discussion 3: Involvement of iron on *Staphylococcus* biofilm formation.

It has been shown that iron regulates biofilm formation of *Staphylococcus* spp. Although the progressive depletion of iron from culture medium has resulted in increasing levels of *S. epidermidis* biofilm production[5], recent studies have demonstrated that iron positively regulates biofilm formation by *Staphylococcus*. For instance, catecholamine inotropes, which remove iron from plasma iron-binding proteins, facilitate the bacterium iron acquisition and stimulate *S. epidermidis* growth as biofilms, suggesting that *S. epidermidis* requires iron to adhere and form biofilms onto surfaces[6].

Since it is noteworthy that some flavonoids and tannins are strong iron chelators, the ability of the PMP to bind iron was investigated using a ferrozine assay (Supplementary Fig. S10a). In this sense, Lin and co-works (2012)[7] revealed that pentagalloylglucose impairs *S. aureus* biofilm formation through Fe^{II} chelation. That iron supplementation compensates the effect of pentagalloylglucose restoring biofilm formation, which illustrates the essential role of Fe^{II} for *S. aureus* biofilm formation. The iron chelator 2,2-bypiridyl, commonly used to deplete free iron in solution, was used as a control. A gradual decrease of ironconcentration bound to ferrozine was observed for 2,2-bypiridyl (Supplementary Fig. S10b). However, the complex ferrozine-Fe^{II} remained unchanged even in the presence of PMP at 4.0 mg mL^{-1} (Fig. 3a), indicating the absence of competition for Fe^{II} between proanthocyanidins and ferrozine. It is an important result since reveals a difficulty of these compounds in establishes an intermolecular interaction with iron and, in addition, it points out the broad effect on surface changes as the major mechanism of action for the anti-adhesive effect showed by PMP against *S. epidermidis*.

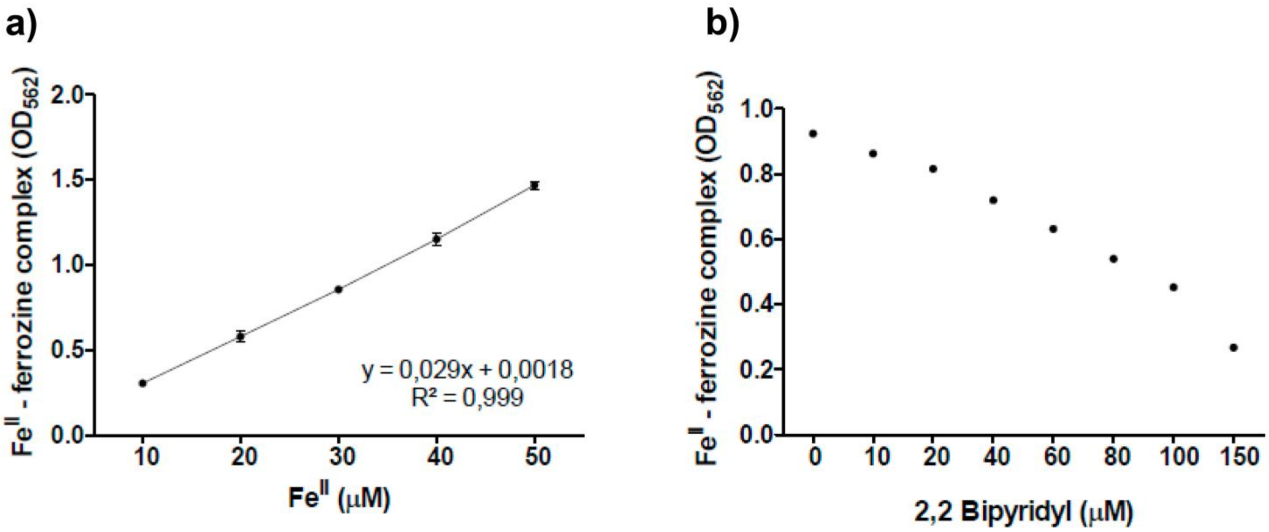

Figure S10 Iron-chelating assessment: (a) The standard curve established to determine the Fe^{II} concentration in a ferrozine complex; (b) Ferrozine-Fe^{II} complex quantified in the presence of positive-chelator 2,2-bipyridyl.

4. Data 2: Assessment viability of planktonic cells and water resistance of the coating.

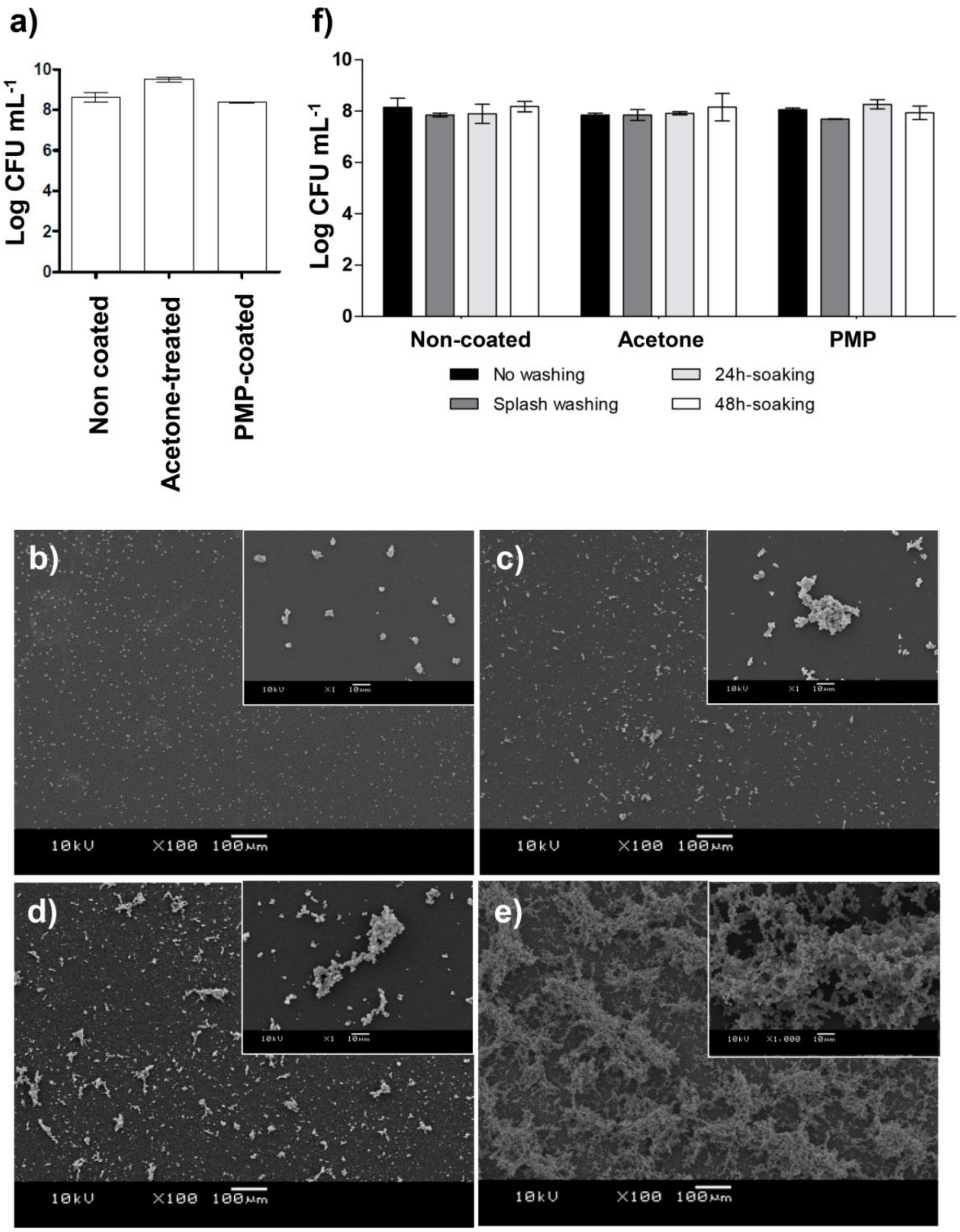

Figure S11 (a) Bacterial viability of planktonic cells from non-treated Permanox; 70% acetone-treated Permanox and PMP coated-Permanox (coated using 70% acetone as diluent), without any washing procedure. (b-e) Bacterial adhesion onto Permanox surface coated with (b) PMP (using 70% acetone as diluent) without any washing procedure; (c) coated with PMPs and followed by splash wash in physiologic solution; (d) coated with PMP and followed by soaking during 24 h in physiologic solution; (e) coated with PMP and followed by soaking during 48 h in physiologic

solution. (f) The viability of *S. epidermidis* on the surface after washing the coating using different procedures. Methodologies can be found in Supplementary Methods

5. Methods

5.1 UPLC-DAD-MS analyses. An ACQUITY UPLC-MS System (Waters Assoc., Milford, USA) and a column ACQUITY 1.7 µm C18 BEH (2.1 mm × 50 mm) were used. The fractions were solubilized in ACN:H_2O (1:1, v/v) at 80 µg mL^{-1}. The mobile phase was ACN (B) and deionized water (A) containing 0.1% formic acid. The column temperature was maintained at 30°C, and the samples were conditioned at 10°C in the automatic injector. The injection volume was 5 µL, the mobile phase flow rate was 0.3 mL min^{-1} and the elution profile was the following: 0-0.9 min, 5% B; 0.9-5.1 min, 5-20% B; 5.1-8.5 min, 20-50% B; 8.5-10.0 min, 50-100% and 10.0-11.0 min, 100% B. MS conditions using both ionization modes (positive and negative) were the following: cone energy of 25 kV; collision energy, 30 eV; and capillary energy, 2.5 kV. Nitrogen was used as nebulizing and drying gas (650 L h^{-1}, 350 °C) and argon was used as the collision gas.

5.2. Pre-treatment of bacterial cells with PMP prior to adhesion and viability assays. A set of experiments were performed by mixing the *S. epidermidis* suspension, proanthocyanidins and TSB in sterile plastic tubes, maintaining the proportions used in the microplates, and incubating for 24 h at 37 °C. Afterwards, the following procedures were performed: (i) bacterial cells were washed three times with sterile 0.9% NaCl solution and harvested by centrifugation in order to obtain a proanthocyanidin-treated bacterial pellet; (ii) each inoculum was adjusted with sterile 0.9% NaCl solution (OD_{600} of 0.150); (iii) these pre-treated cells were evaluated for biofilm formation and bacterial viability by crystal violet assay and by counting of CFU mL^{-1}, respectively.

5.3 Bacterial growth and viability. Bacterial growth was evaluated in the microplates by measuring the difference between the arithmetic means of the OD_{600} of the cultures after 24 h (incubation time) in wells containing the proanthocyanidins, TSB and bacterial suspension, from the wells containing the proanthocyanidins, TSB and sterile saline (without inoculum); thus, the

interference of the sample color in the results obtained by OD evaluations was avoided. As controls for bacterial growth, the proanthocyanidins were replaced by water or by rifampicin (Sigma-Aldrich Co., USA). To verify the viability of bacterial cells, serial dilutions of the wells were performed and were spread on MH agar plates. After overnight incubation at 37 °C, the number of colony-forming units (CFU) was determined and expressed as log CFU mL^{-1}.

5.4 Scanning Electron Microscopy. Biofilms of *S. epidermidis* were grown in 96-well microtiter plates, with a piece of Permanox slide or a glass coverslip in each well. After 24 h at 37 °C, the samples were fixed in 2.5% glutaraldehyde, washed with 100 mM cacodylate buffer pH 7.2 and dehydrated in increasing concentrations of acetone. The samples were dried using the CO_2 critical point technique and examined in a scanning electron microscope.

5.5 Confocal Laser Scanning Microscopy. Using sterile polystyrene and coverglass-bottom dishes, bacterial suspension, proanthocyanidins and TSB were incubated (24 h at 37 °C). The samples were then stained with LIVE/DEAD BacLight Bacterial Viability Kit (Life Technologies, USA) for 30 minutes at room temperature in darkness, and the dish contents were gently replaced with water. Finally, samples were directly observed using a confocal laser scanning microscope (Leica DM 6000 CS) with a 63X upright objective. For each sample, a sequential scan in two channels was carried out and corresponding xy optical sections and 4 random images were acquired. Overlapping images and orthogonal cuts were obtained by using MetaMorph and Imaris x64 software.

5.6 Iron chelating assessment. To evaluate whether iron chelation could account for proanthocyanidins activity against *S. epidermidis* biofilm formation, the ferrozine assay was performed using Fe^{II} as source of iron, as reported in previous studies[7], since any Fe^{III} can be reduced to Fe^{II} in presence of polyphenols[8,9], which was also experimentally confirmed. A standard curve was established (Supplementary Fig. S10a) to determine the Fe^{II} concentration to be used in the ferrozine assay as described elsewhere[9]. Formation of the iron-ferrozine complex was

measured at 562 nm to assess the amount of iron chelated by proanthocyanidins. For comparison of iron-chelating activity, 2,2-bipyridyl (Sigma-Aldrich Co., USA) was used as a standard iron chelator.

5.7 Bacterial surface hydrophobicity index. Surface hydrophobicity of *S. epidermidis* was determined using the microbial adhesion to hydrocarbon (MATH) test[10]. After treatment with proanthocyanidins, bacterial cells were washed and harvested by centrifugation. The bacterial suspensions were adjusted to an initial OD_{600} (OD_i) of about 0.3. Toluene was added to each adjusted bacterial suspension and mixed. The final absorbances of the aqueous phase (OD_f) were measured after phase separation. The hydrophobicity index (HPBI) was expressed as equation (1): $(OD_i - OD_f) / OD_i \times 100\%$. Values of HPBI greater than 70% indicated a hydrophobic bacterial surface and lesser than 70% indicated a hydrophilic bacterial surface.

5.8 Non-biological model particle adhesion experiments. Experiments using red fluorescent FluoSpheres™ beads (Life Technologies, USA), which present hydrophobic and anionic surface and similar size as the *S. epidermidis* (1.0 μm diameter), were conducted using the same concentration as the bacterial suspension (3×10^8 particles mL^{-1}). These experiments were performed using sterile polystyrene and coverglass-bottom dishes according CLSM experiments, replacing bacterial cells with the microspheres. The dishes were incubated at 37 °C for 24 h and then, they were gently washed with sterile 0.9% NaCl and immediately visualized in using an Olympus IX71 fluorescence microscope.

5.9 Water resistance of the coating. The water resistance of the PMP coating was tested using three different washing regimens: i) by splash-wash or ii) by soaking the coated specimens in sterile 0.9% NaCl (saline solution) at room temperature, in two different times. i) In the splash-wash the samples were kept upright in individual 15 mL-sterile tubes, where sterile saline was added, immediately aspired and pippeted out. This procedure was repeated 30 times, in order to mimic friction of coating with water. ii) The soaking procedure was conducted in individual 15 mL

sterile tubes filled with the sterile saline to a depth such that the test specimens were completely immersed. The samples were kept immersed in the solution for 24 and 48 h. After the splash or soaking washes, all samples were evaluated according their ability to prevent *S. epidermidis* adhesion and biofilm formation and to preserve the viability of bacterial cells.

6. References

1. Markham, K.R. In *Techniques of Flavonoid Identification*, Markham K.R., Ed.; Academic Press: London, 1982; p. 36-51.

2. Fabre, N., Rustan, I., Hoffmann, E. & Quetin-Leclercq, J. Determination of flavone, flavonol, and flavanone aglycones by negative ion liquid chromatography electrospray ion trap mass spectrometry. *J. Am. Soc. Mass Spectrom*. **12,** 707-715 (2001).

3. March, R. E, Lewars, E. G., Stadey, C. J., Miao, X. S., Zhao, X. & Metcalfe, C. D. A comparison of flavonoid glycosides by electrospray tandem mass spectrometry. *Int. J. Mass Spec*. **248,** 61-85 (2006).

4. Scmidt, C. A., Murillo, R., Heinzmann, B., Laufer, S., Wray, V. & Merfort, I. Structural and conformational analysis of proanthocyanidins from *Parapiptadenia rigida* and their wound-healing properties. *J. Nat. Prod*. **74,** 1427-1436 (2011).

5. Deighton, M. & Borland, R. Regulation of slime production in *Staphylococcus epidermidis* by iron limitation. *Infect. Immun.* **61,** 4473-4479 (1993).

6. Lyte, M. *et al*. *Stimulation of Staphylococcus epidermidis growth and biofilm formation by catecholamine inotropes. Lancet* ***361,*** *130-135 (2003).*

7. Lin, M. H., Shu, J. C., Huang, H. Y. & Cheng, Y. C. Involvement of iron in biofilm formation by *Staphylococcus aureus*. *PLoS One* **7,** e34388 (2012).

8. Guo, M. Iron-binding properties of plant phenolics and cranberry's bioeffects. *Dalton Trans*. **43**, 4951–4961 (2007).

9. Mladenka, P*. et al*. *In vitro* analysis of iron chelating activity of flavonoids. *J. Inorg. Biochem.* **105**, 693-701 (2011).

10. Nostro, A. *et al*. Modifications of hydrophobicity, in vitro adherence and cellular aggregation of *Streptococcus mutans* by *Helichrysum italicum* extract. *Lett. Appl. Microbiol.* **38**, 423-427 (2004).
